# Multi-heterointerfaces for selective and efficient urea production

**DOI:** 10.1093/nsr/nwac209

**Published:** 2022-10-04

**Authors:** Danyan Zhang, Yurui Xue, Xuchen Zheng, Chao Zhang, Yuliang Li

**Affiliations:** Institute of Chemistry, Chinese Academy of Sciences, Beijing 100190, China; University of Chinese Academy of Sciences, Beijing 100049, China; Institute of Chemistry, Chinese Academy of Sciences, Beijing 100190, China; Shandong Provincial Key Laboratory for Science of Material Creation and Energy Conversion, Science Center for Material Creation and Energy Conversion, School of Chemistry and Chemical Engineering, Shandong University, Jinan 250100, China; Institute of Chemistry, Chinese Academy of Sciences, Beijing 100190, China; University of Chinese Academy of Sciences, Beijing 100049, China; Institute of Chemistry, Chinese Academy of Sciences, Beijing 100190, China; University of Chinese Academy of Sciences, Beijing 100049, China; Institute of Chemistry, Chinese Academy of Sciences, Beijing 100190, China; Shandong Provincial Key Laboratory for Science of Material Creation and Energy Conversion, Science Center for Material Creation and Energy Conversion, School of Chemistry and Chemical Engineering, Shandong University, Jinan 250100, China; University of Chinese Academy of Sciences, Beijing 100049, China

**Keywords:** porous materials, carbon materials, multi-heterointerfaces, urea synthesis, high-performance conversion

## Abstract

A major impediment to industrial urea synthesis is the lack of catalysts with high selectivity and activity, which inhibits the efficient industrial production of urea. Here, we report a new catalyst system suitable for the highly selective synthesis of industrial urea by *in situ* growth of graphdiyne on the surface of cobalt–nickel mixed oxides. Such a catalyst is a multi-heterojunction interfacial structure resulting in the obvious incomplete charge-transfer phenomenon between a graphdiyne and metal oxide interface and multiple intermolecular interactions. These intrinsic characteristics are the origin of the high performance of the catalyst. Studies on the mechanism reveal that the catalyst could effectively optimize the adsorption/desorption capacities of the intermediate and promote direct C–N coupling by significantly suppressing by-product reactions toward the formation of H_2_, CO, N_2_ and NH_3_. The catalyst can selectively synthesize urea directly from nitrite and carbon dioxide in water at room temperature and pressure, and exhibits a record-high Faradaic efficiency of 64.3%, nitrogen selectivity (N_urea_-selectivity) of 86.0%, carbon selectivity (C_urea_-selectivity) of ∼100%, as well as urea yield rates of 913.2 μg h^−1^ mg_cat_^−1^ and remarkable long-term stability.

## Teaser

A multiple heterogeneous interface with unique incomplete charge transfer is constructed by growing graphdiyne *in situ* on cobalt–nickel mixed oxides surface, effectively promoting high-performance urea production at room temperature and pressure.

## INTRODUCTION

Urea [CO(NH_2_)_2_] has always influenced the development of industry and agriculture in the world because of its important position in the agriculture and chemical industry [1,2]. The development of efficient urea production is of great significance for increasing crop production to meet the demands of a growing population and basic industrial raw materials [3,4]. However, current industrial urea production is mainly achieved by (i) reacting liquid ammonia (NH_3_) with liquid carbon dioxide (CO_2_) to form ammonium carbamate (NH_2_COONH_4_) and (ii) the decomposition of NH_2_COONH_4_ to obtain urea and water at high temperatures and pressures consuming large amounts of fossil fuels [5–7]. In addition, the raw material NH_3_ is produced by a hashing, energy-intensive, complex Haber–Bosch process [8,9]. In order to alleviate the problem of energy shortage and achieve the goal of carbon neutrality, exploring catalyst innovation development is a new path for the sustainable development of the world economy in the future [10–13].

Electrochemical coupling of N_2_ and CO_2_ in water for urea synthesis is an attractive approach [14,15]. However, the high activation energy barrier of N≡N and the limited solubility of N_2_ in water lead to a low urea yield rate (*Y*_urea_) and low Faradaic efficiency (FE) [16–18]. In view of this, exploration of the coupling of NO_2_^−^ with CO_2_ in water with high solubility and a low activation energy barrier may provide a renewable and economically promising route for urea production under ambient conditions [19,20]. As expected, achieving high FE and nitrogen atomic efficiency (NE) in urea simultaneously is very desirable, but this is an acknowledged scientific challenge that must be overcome by the following factors: how to control the competition of the parallel CO_2_ and/or NO_2_^−^ reduction and hydrogen evolution reactions resulting in low FE, how to improve the low selectivity for the C–N coupling and how to improve the selectivity to form intermediates of reaction.

Catalysts with multi-heterointerface structures generally show higher selectivity and activity in catalysis than single-component ones due to the improved electron transferability, the unevenly distributed interface charge on the surface of the catalysts, the increased number of active sites and the optimized adsorption/desorption behaviors of the reactants/intermediates [21–25]. A key strategy for building such an interface is the perfect combination of multicomponent nanoparticles (e.g. metal oxides, hydroxides or metal alloys) as acceptor units with supporting materials as donor units [26–28]. Such catalysts greatly improve the selectivity, activity and stability compared with single-component ones, due to high-density charge transfer between donors and receptors [29–31]. This provides a solid foundation for our rational design of high-performance multi-interface catalysts.

Graphdiyne (GDY), a rising star on the horizon of carbon materials comprising sp/sp^2^-cohybridized carbon atoms, has established a solid position in the fields of electrocatalysis, photocatalysis, energy conversion, etc. [32–35] due to its fascinating and unique advantages such as the uneven surface charge distribution, infinite natural pores, highly π-conjugated structure, excellent stability, etc. Notably, GDY is the only all-carbon material that can be grown in arbitrary materials, which allows the controlled synthesis of ideal interface structures with determined valence states and accurate chemical structures [36–38]. Besides, the unique incomplete charge-transfer ability of GDY-based catalysts endows the catalysts with ultra-high catalytic selectivity and activity for various reactions [39,40].

In this study, selective and efficient urea production was achieved on a multi-heterojunctions interfacial structure of Co–NiO_x_@GDY. Experimental results show that the unique structures of the catalyst can result in the strong incomplete charge-transfer phenomenon between the GDY and metal oxide interface and multiple intermolecular interactions, leading to high electrocatalytic performances. Studies on the mechanism show that Co–NiO_x_@GDY can simultaneously optimize the CO_2_/CO adsorption ability and promote the NH_3_ formation, which is expected to provide large abundant _*_CO intermediates and NH_2_-related intermediates for the direct C–N coupling accompanied by the significant suppression of the by-product reactions toward the formation of H_2_, CO, N_2_ and NH_3_. Benefitting from these unique features, Co–NiO_x_@GDY with multi-heterointerfaces reaches a record-high FE of 64.3%, N_urea_-selectivity of 86.0%, C_urea_-selectivity of ∼100%, as well as urea yield rates of 913.2 μg h^−1^ mg_cat_^−1^ and remarkable long-term stability.

## RESULTS AND DISCUSSION

**Figure 1.**
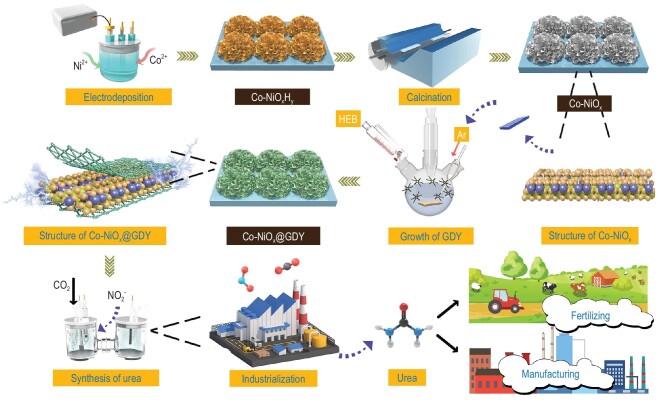
Schematic representation of the synthesis routes of Co–NiO_x_@GDY.

Figure 1 illustrates the synthesis route for the controlled synthesis of Co–NiO_x_@GDY through a three-step strategy including the first growth of a film of cobalt–nickel bimetal mixed layered double hydroxide nanosheets on the surface of nickel foam (Co–NiO_x_H_y_) via an electrodeposition method, followed by a calcination treatment of the as-prepared Co–NiO_x_H_y_ at 300°C for 2 h during which the porous Co–NiO_x_ nanosheets were obtained, and finally the *in situ* growth of ultra-thin GDY films on the surface of Co–NiO_x_ through a cross-coupling reaction with hexaethynylbenzene (HEB) as the precursor (please see the Experimental Section for more details).

**Figure 2.**
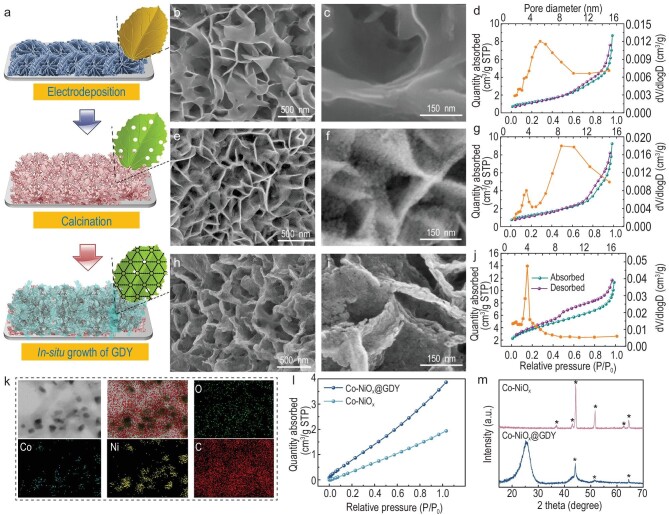
(a) Model diagram of the catalysts. (b) Low- and (c) high-magnification SEM images of Co–NiO_x_H_y_. (d) The N_2_ adsorption/desorption isotherms and pore-size distributions of Co–NiO_x_H_y_. (e) Low- and (f) high-magnification SEM images of Co–NiO_x_. (g) The N_2_ adsorption/desorption isotherms and pore-size distributions of Co–NiO_x_. (h) Low- and (i) high-magnification SEM images of Co–NiO_x_@GDY. (j) The N_2_ adsorption/desorption isotherms and pore-size distributions of Co–NiO_x_@GDY. (k) The STEM and elemental mapping images of Co, Ni, C and O in the Co–NiO_x_@GDY. (l) The CO_2_ adsorption isotherms comparison of Co–NiO_x_ and Co–NiO_x_@GDY. (m) Powder XRD patterns of Co–NiO_x_ and Co–NiO_x_@GDY.

The models in Fig. 2a)illustrate the morphology changes of the samples from nanosheets with smooth surfaces to porous ones and finally to multilayered 2D nanosheets. As shown in Fig. 2b)and c, a film of Co–NiO_x_H_y_ nanosheets with a wrinkled surface was vertically aligned and ordered grown on the surface of the substrates. A 3D porous electrode with uniform element distribution was then obtained (Supplementary Fig. S1). Such architectures benefit from the increase in the surface area and the number of active sites of the samples. After the calcination treatment, the Co–NiO_x_ nanosheets collapsed and became more porous due to the dehydration of the precursors at high temperatures (Fig. 2e)and f, and Supplementary Fig. S2), resulting in a larger specific surface area (SSA) of 4.68 m^2^ g^−1^ (Fig. 2g) than the Co–NiO_x_H_y_ sample (Fig. 2d). By using Co–NiO_x_ nanosheets as the substrate, the GDY nanosheets were further *in situ* grown on the surface of the Co–NiO_x_ nanosheets (Fig. 2h)and i), leading to the formation of a vertically aligned and densely interconnected ordered 3D electrode. Figure 2k)shows the uniform distribution of Co, Ni, O and C elements for the Co–NiO_x_@GDY sample, revealing the successful growth of GDY. The absence of a Cu signal in the total spectrum of the elemental distribution surface (Supplementary Fig. S3) proves that our synthesized material does not contain Cu elements and successfully avoids the interference of Cu elements in the catalytic reaction.

The mesoporous nature of the prepared materials was then identified from the nitrogen adsorption–desorption isotherm at 77 K. Co–NiO_x_H_y_ (Fig. 2d) and Co–NiO_x_ (Fig. 2g) samples show Type IV isotherms with an H_3_-type hysteresis loop [41,42], which indicates the presence of a mesoporous structure with mesopores predominantly lying between 5–7 and 8–10 nm, respectively. Co–NiO_x_@GDY has an H_4_-type hysteresis loop with a more pronounced hysteresis loop (Fig. 2j). Correspondingly, the pores in Co–NiO_x_@GDY show smaller sizes concentrated at 4 nm than Co–NiO_x_H_y_ (Fig. 2d) and Co–NiO_x_ (Fig. 2g). As expected, Brunauer–Emmett–Teller results show that Co–NiO_x_@GDY has the largest SSA of 13.70 m^2^ g^−1^ compared with the Co–NiO_x_H_y_ and Co–NiO_x_ samples, indicating the presence of more active sites in the presence of GDY. Co–NiO_x_@GDY also shows a much higher CO_2_ uptake ability (3.86 cm^3^ g^−1^) at 298 K than pure Co–NiO_x_ (1.93 cm^3^ g^−1^), indicating the greatly enhanced CO_2_ affinity after the introduction of GDY. XRD patterns for Co–NiO_x_ (Fig. 2m) show two diffraction peaks at 36.61° and 44.54° corresponding to the (311) and (400) crystal planes of NiCo_2_O_4_, one diffraction peak at 42.8° corresponding to the (100) crystal plane of NiCoO_2_, the peak at 62.85° corresponding to the (110) crystal plane of NiO and the peak at 51.2° corresponding to the (100) crystal of Co, respectively [43]. After the *in situ* growth of GDY, the intensity of the metal peaks decreased and new peaks (at 25.42° and 43.14°) corresponding to the carbon materials (GDY) were observed, which indicates the successful incorporation of GDY and Co–NiO_x_ species.

**Figure 3.**
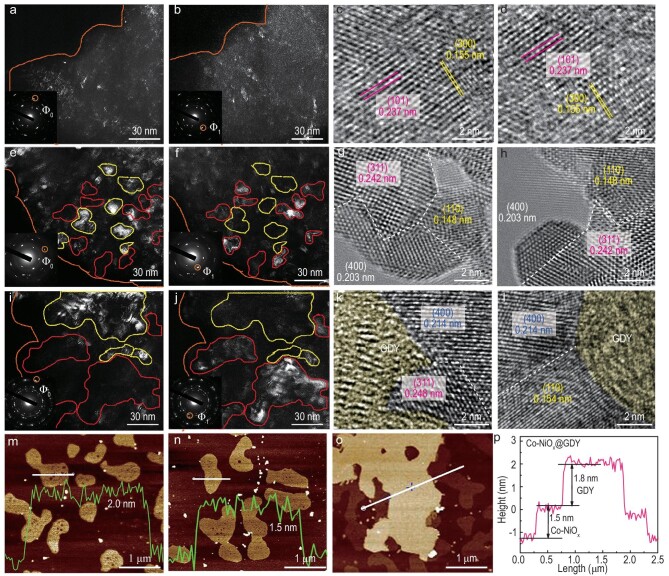
(a) DF-TEM images of Co–NiO_x_H_y_, obtained by selecting the inner diffraction spots Φ_o_ [corresponding to Ni(OH)_2_ (300) crystal diffraction lattice]. (b) DF-TEM images of Co–NiO_x_H_y_, obtained by selecting the inner diffraction spots Φ_1_ [corresponding to Co(OH)_2_ (101) crystal diffraction lattice]. (c and d) The HR-TEM images of Co–NiO_x_H_y_. (e) DF-TEM images of Co–NiO_x_, obtained by selecting the inner diffraction spots Φ_o_ [corresponding to NiO (110) crystal diffraction lattice]. (f) DF-TEM images of Co–NiO_x_, obtained by selecting the inner diffraction spots Φ_1_ [corresponding to Co–NiO_x_ (400), (311) crystal diffraction lattice]. (g and h) The HRTEM images of Co–NiO_x_. (i) DF-TEM images of Co–NiO_x_, obtained by selecting the inner diffraction spots Φ_o_ [corresponding to NiO (110) crystal diffraction lattice]. (j) DF-TEM images of Co–NiO_x_, obtained by selecting the inner diffraction spots Φ_1_ [corresponding to Co–NiO_x_ (400), (311) crystal diffraction lattice]. (k and l) The HRTEM images of Co–NiO_x_@GDY. The AFM images and thickness measurement of (m) Co–NiO_x_H_y_, (n) Co–NiO_x_ and (o and p) Co–NiO_x_@GDY.

Transmission electron microscopy (TEM) images show the presence of Ni(OH)_2_ (300), Co(OH)_2_ (101) in Co–NiO_x_H_y_ nanosheets(Supplementary Fig. S4). Dark-field TEM (DF-TEM) is a high-throughput and diffraction-sensitive imaging technique, which can directly image crystal symmetry by selecting an inner diffraction spot with a selected aperture in the diffraction pattern [44–47]. We use selected-area electron diffraction and DF-TEM to characterize the crystal structure of our catalysts. The bright-field image and diffraction pattern (Supplementary Fig. S5) reveal that Co–NiO_x_H_y_ contains several grains with different orientations. Figure 3a)and b shows the corresponding DF-TEM images from two different areas with different selected spots in the diffraction pattern. Obviously, the hydroxide Co–NiO_x_H_y_ obtained by direct electrodeposition with poor crystallinity results in poor visualization of the directional diffraction. The high-resolution TEM (HRTEM) images of Co–NiO_x_H_y_ (Fig. 3c)and d) show nanosized crystallites with various orientations, including the (300) plane of Ni(OH)_2_ with a lattice constant of 0.155 nm and the (101) plane of Co(OH)_2_ with a lattice constant of 0.237 nm, match well with the XRD results (Supplementary Fig. S6). After calcination, the Co–NiO_x_ nanoarrays are porous with a more rigid and tighter structure (Supplementary Fig. S7a and b).

The bright-field image and diffraction pattern of the as-prepared Co–NiO_x_ revealed the polymorphism of the Co–NiO_x_ (Supplementary Fig. S8). DF-TEM images in Fig. 3e)and f reveal that the samples are constituted by the NiO (110) facets and Co–NiO_x_ (400) and (311) facets, respectively, accompanied by numbers of grain boundaries that might lead to the formation of new active sites. When overlaying the two dark-field images, we marked the highlighted area in the NiO (110) dark-field image as yellow and the Co–NiO_x_ (400), (311) dark-field image as red. In the superimposed dark-field images, we found that the yellow area and the red area show no overlaps but only one kind of grain appears in the same position, which proves that Co–NiO_x_ is multiphased. For a deeper understanding of the Co–NiO_x_ grains, the HRTEM images in Fig. 3g)and h depict mainly nanosized grains with three different orientations. We identified diverse geometries for individual grains and the (110) plane of NiO; the (311) and (400) planes can be clearly observed with lattice constants of 0.148, 0.242 and 0.203 nm, respectively. Co–NiO_x_ contains a high density of grain interfaces, which causes the distortion of the atomic layers at the interface (Supplementary Fig. S9) and a high density of dislocations and steps, which are helpful to expose additional catalytic sites and improve the catalytic performance [48]. Such a unique grain boundary enriched structure is well preserved after the *in situ* growth of GDY nanosheets in the TEM images of Co–NiO_x_@GDY (Supplementary Fig. S10a and b) in which some tiny crystallite fragments have coalesced to form larger ones. Supplementary Fig. S11 shows the bright-field image and diffraction pattern of the as-prepared Co–NiO_x_@GDY. The larger highlighted regions in DF-TEM images (Fig. 3i)and j) indicate that some of the tiny grains have coalesced to form large grains with a clear boundary. This is well characterized by the HRTEM in Fig. 3k)and l. Compared with pristine Co–NiO_x_, the lattice constant in Co–NiO_x_@GDY increases at the interface between the GDY and the edge of the Co–NiO_x_. Generally, the lattice constant of the (400) plane of Co–NiO_x_ increases from 0.203 to 0.214 nm, accompanied by the increase in the (311) plane of Co–NiO_x_ from 0.242 to 0.248 nm. Meanwhile, the lattice constant of the (110) plane of NiO increases from 0.148 to 0.254 nm. As clearly observed in the HRTEM images (Fig. 3l), many disordered domains were formed at the interface between the GDY and Co–NiO_x_ species after the *in situ* growth of the GDY on the Co–NiO_x_ surface, which are beneficial for regulating the local electronic structures and coordination environments, and beneficial for improving the catalytic activity of the samples (Supplementary Fig. S12) [49–51]. Besides, a specific characteristic crystallization pattern of the GDY with an interplanar distance of 0.465 nm can be revealed which matches that of the ABC stacking mode (Supplementary Fig. S10c). The AFM results show that the Co–NiO_x_H_y_ nanosheets have a thickness of ≈2.0 nm (Fig. 3m)and Supplementary Fig. S13), whereas the Co–NiO_x_ nanosheets have a thickness of ≈1.5 nm (Fig. 3n)and Supplementary Fig. S14). Excitingly, after *in situ* growth of the GDY, the AFM image clearly reveals two different heights of nanosheets (Fig. 3p) and a clear step-like change in the thickness measurement represented in Fig. 3p)clearly identifies the superposition of a 1.5-nm Co–NiO_x_ layer and a 1.8-nm GDY layer.

**Figure 4.**
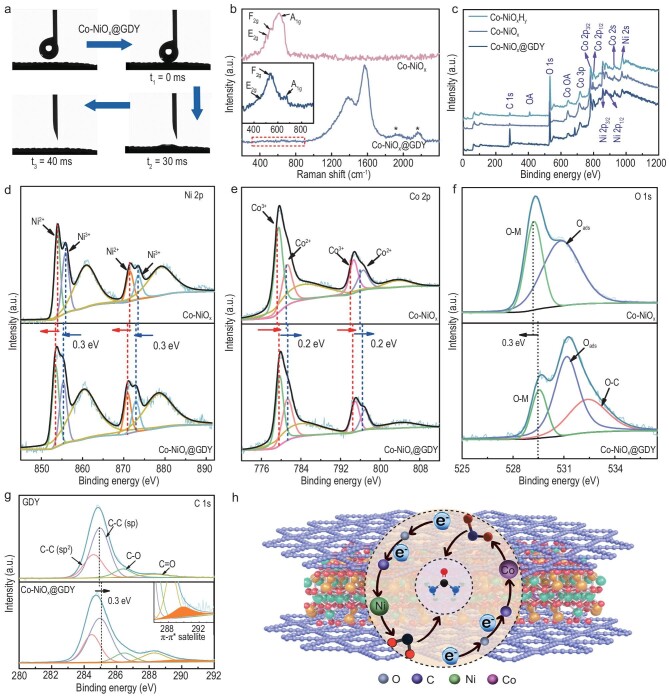
(a) Contact angle measurements of Co–NiO_x_@GDY. (b) Raman spectra of Co–NiO_x_ and Co–NiO_x_@GDY. (c) XPS survey spectra of Co–NiO_x_H_y_, Co–NiO_x_ and Co–NiO_x_@GDY samples. The high-resolution (d) Ni 2p, (e) Co 2p and (f) O 1s XPS spectra of Co–NiO_x_ and Co–NiO_x_@GDY, respectively. (g) The high-resolution C 1s XPS spectra of GDY and Co–NiO_x_@GDY. Inset in (g): the π–π* transition peak of Co–NiO_x_@GDY. (h) Schematic representation of the charge transfer of the multi-heterojunction interfacial structure.

Contact angle measurements (Fig. 4a) showed that Co–NiO_x_@GDY has a super hydrophilic surface with a contact of 0° (Supplementary Fig. S15). Raman and XPS measurements were further performed to study the structure of the catalysts. As shown in Fig. 4b, the peak at 473 cm^−1^ could be attributed to the stretching vibrations of the Co–O and Ni–O bonds in the E_2 g_ Raman active mode; the peaks at 519 and 616 cm^–1^ can be indexed to the F_2 g_ and A_1 g_ Raman active modes of the Co–O stretching vibration of the Co–NiO_x_ sample, respectively [52]. There are no peaks corresponding to the OH group that could be observed from the Raman spectra, indicating the complete formation of the Co–Ni oxide phase after the calcination treatment. For Co–NiO_x_@GDY, four characteristic peaks corresponding to the D band (1385.4 cm^−1^) and G band (1568.5 cm^−1^) and the vibrations of the conjugated diyne links (1934.5 and 2170.3 cm^−1^) were observed [53]. The magnified Raman spectra demonstrated that the signal of Co–NiO_x_ still remains after the *in situ* growth of graphdiyne (Supplementary Fig. S16). Moreover, the XPS survey spectra for the samples (Fig. 4c) confirmed the presence of Ni, Co and O elements in Co–NiO_x_H_y_ and Co–NiO_x_ while an additional C signal could be observed in Co–NiO_x_@GDY. These results solidly confirmed the successful growth of GDY on the surface of the mixed metal oxides, indicating the successful construction of multi-heterojunction interfacial structures. The Ni 2p XPS spectra (Fig. 4d) were well fitted with two spin–orbit doublets and two shakeup satellites (denoted as ‘Sat.’). For Co–NiO_x_, the Ni 2p peaks at 854.0/871.5 and 855.9/873.5 eV are characteristic for the Ni^2+^ and Ni^3+^ species, respectively. The satellite peaks at ∼860.4 and ∼879.8 eV represented shakeup-type peaks of nickel at the high binding energies of Ni 2p_3/2_ and Ni 2p_1/2_. After the *in situ* growth of graphdiyne, the Ni species showed a slight negative shift of 0.3 eV compared with that in Co–NiO_x_. The ratio of Ni^3+^/Ni^2+^ is calculated as 0.919 for Co–NiO_x_, while the ratio of Ni^3+^/Ni^2+^ is calculated as 0.810 for Co–NiO_x_@GDY with a slight decline (Supplementary Fig. S17). The decrease in the ratio of Ni^3+^/Ni^2+^ is consistent with the negative shift of Ni 2p, which demonstrates the electron-withdrawing property of Ni species in the catalyst. The Co 2p XPS spectrum (Fig. 4e) was also fitted with two spin–orbit doublets and two shakeup satellites at ∼802.8 and ∼786.5 eV. For Co–NiO_x_, the binding energies at 779.5 and 794.7 eV can be ascribed to Co^3+^ species and the others at 781.2 and 796.6 eV were ascribed to Co^2+^ species. After the *in situ* growth of graphdiyne, the Co species showed a slight positive shift of 0.2 eV compared with that in Co–NiO_x_. In addition, for Co–NiO_x_, the ratio of Co^3+^/Co^2+^ is calculated as 1.559 while the ratio of Co^3+^/Co^2^ is calculated as 1.396 for Co–NiO_x_@GDY with a slight decline (Supplementary Fig. S18). The decrease in the ratio of Co^3+^/Co^2^ is consistent with the result that the positive shift of Co 2p, which demonstrates the electron-withdrawing property of Co species in the catalyst. The above-discussed XPS results demonstrate that the Co, Ni species in the catalyst possess mixed valence states, which has been demonstrated to enhance the catalytic activity. The O 1s XPS spectra (Fig. 4f) for Co–NiO_x_ showed two peaks of metal–O (529.2 eV) and adsorbed oxygen (530.8 eV). For Co–NiO_x_@GDY, the newly formed peak at 532.4 eV could be ascribed to the C–O bonds between the O elements in Co–NiO_x_ and the C elements in GDY. This also indicate the formation of the ‘C–O–metal’ structures at the heterointerfaces between Co–NiO_x_ and GDY, which benefits the formation of new catalytic active sites [54]. As shown in Fig. 4g, four sub-peaks corresponding to the C–C (sp^2^, 283.9 eV), C–C (sp, 284.9 eV), C–O (286.3 eV) and C=O (288.2 eV) were observed for pristine GDY. For Co–NiO_x_@GDY, in addition to the characteristic peaks for C–C (sp^2^, 283.9 eV), C–C (sp, 285.2 eV), C–O (286.4 eV) and C=O (288.4 eV), an additional π–π* satellite peak at 289.95 eV was observed, indicating the interactions between GDY and Co–NiO_x_ species. The intensity ratio of C–C (sp^2^) to C–C(sp) for Co–NiO_x_@GDY remains at 0.5, demonstrating the successful growth of GDY on the Co–NiO_x_ surface. The positive shifts in the binding energies in the sp–C peak indicate the electron-donating property of GDY. These results demonstrated the successful construction of the heterojunction interfacial donor–acceptor structures with incomplete electron transfer between Co–NiO_x_ species and GDY. Moreover, the presence of mixed nickel–cobalt oxidation states in Co–NiO_x_@GDY also can promote the electron transfer between Co–NiO_x_ and GDY, leading to great enhancement in the catalytic ability [55–57].

**Figure 5.**
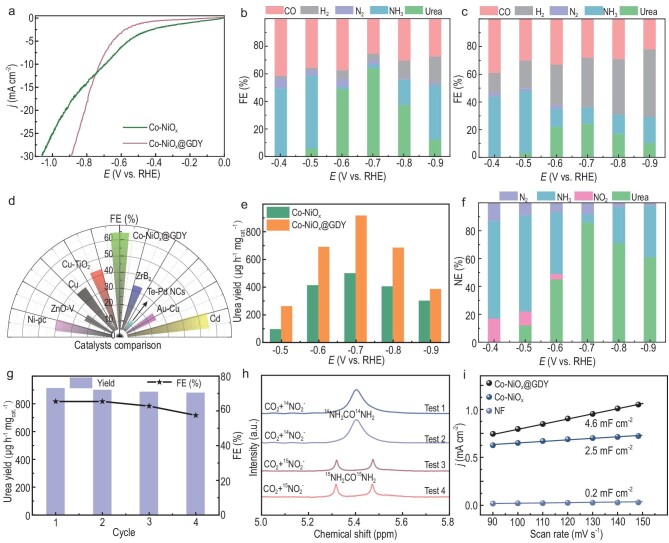
(a) Linear sweep voltammetry curves for Co–NiO_x_ and Co–NiO_x_@GDY in CO_2_-saturated 0.01 M NaNO_2_ solution at a scan rate of 2 mV s^−1^. FE obtained at different potentials for (b) Co–NiO_x_@GDY and (c) Co–NiO_x_. (d) Comparison of the FE value of Co–NiO_x_@GDY with reported catalysts. (e) Urea yield rates of Co–NiO_x_ and Co–NiO_x_@GDY obtained at different potentials. (f) N_urea_-selectivity at different potentials for Co–NiO_x_@GDY. (g) Stability tests for continuous generation of urea. (h) ^1^H NMR spectra of the electrolyte obtained in ^15^N-Isotope labeling experiments. (i) The capacitive currents plotted against the scan rates from 90 to 150 mV s^−1^.

The electrocatalytic performance of the as-synthesized catalysts toward urea production was studied in an H-type electrolytic cell at ambient temperatures and pressures (Supplementary Fig. S19). CO_2_ gas continuously flowed into the cathodic electrolyte containing 0.01 M NaNO_2._ The concentration of the produced urea was measured using the diacetyl monoxime method (Supplementary information). Figure 5a)reveals that the onset potential of Co–NiO_x_@GDY is much more positive and the current density is higher than that of pristine Co–NiO_x_. Co–NiO_x_@GDY with optimized contents of 3.51 wt% achieved the maximum FE value of 64.3% (Supplementary Fig. S20 and Supplementary Table S1) at a low applied potential of −0.7 V versus RHE (Fig. 5b), which is almost three times higher than that of Co–NiO_x_ at 24.3% (Fig. 5c)and Supplementary Fig. S21) and 15 times higher than that of pure GDY at 4.42% (Supplementary Fig. S22) and much higher than all reported electrocatalysts, such as Ni–Pc (40%) [58], Cu–TiO_2_ (43.1%) [59], Te–Pd NCs (12.2%) [19] and ZnO–V (23.3%) [60] (Fig. 5d). It is worth mentioning that the electrolyte concentration is optimized for maximum FE (Supplementary Fig. S23). It was observed that the FE for urea production decreased with the further increase in the applied potentials, which might be due to the interference of the side reactions. In order to determine the specific role of each part in the electrocatalysts in promoting urea synthesis, samples of NiO, CoO, Co–NiO_x_, NiO@GDY and CoO@GDY were prepared (Supplementary Fig. S24). The catalytic performances of these samples have also been measured in urea synthesis (Fig. 5e)and Supplementary Fig. S25). Co–NiO_x_@GDY shows the best catalytic performance with the highest FE (64.3%) and urea yield rates (Y_urea_, 913.2 μg h^−1^ mg_cat_^−1^) compared with that of NiO (FE = 6.6%; Y_urea_ = 221.9 μg h^−1^ mg_cat_^−1^), CoO (FE = 14.3%; Y_urea_ = 217.9 μg h^−1^ mg_cat_^−1^), Co–NiO_x_ (FE = 24.3%; Y_urea_ = 497.2 μg h^−1^ mg_cat_^−1^), NiO@GDY (FE = 16%; Y_urea_ = 221.9 μg h^−1^ mg_cat_^−1^) and CoO@GDY (FE = 20.4%; Y_urea_ = 356.3 μg h^−1^ mg_cat_^−1^). It was found that the catalytic performances of the samples with multiple components were better than those with single components, which might be due to the formation of mixed valence states of the metal species and the grain boundary dislocations in the multicomponent sample. Besides, the introduction of GDY can greatly improve the catalytic activity of the samples due to the formation of strong incomplete charge transfer between GDY and the metal atoms at the multi-heterojunction interface, which can significantly improve the conductivity, increase the number of active sites and finally enhance the overall electrocatalytic performances of the electrocatalyst for urea synthesis. Based on above discussion, the strong incomplete charge transfer between the GDY and metal oxide interface that occurred on the multi-heterojunction interface structure of the electrocatalyst plays a critical role in enhancing the catalytic performance for urea synthesis. Interestingly, the amounts of CO during the urea production process in nitrite-containing electrolytes were obviously lower than those absent of nitrite at all potentials (Supplementary Fig. S26), which indicates that the resulted _*_CO from CO_2_ reduction mainly participates in the C–N coupling reaction forming urea and simultaneously inhibits the parallel competition reactions, which can greatly increase the FE of the reaction. In order to precisely determine the distribution of N species during the urea production process, the NE results were calculated (Fig. 5f). When compared to the electrolyte without CO_2_, the conversion of NO_2_^−^ and the production of NH_3_ are increased in CO_2_-saturated electrolytes (Supplementary Fig. S27). Co–NiO_x_@GDY shows the maximum urea NE of 86.2% at −0.7 V versus RHE, which indicates that almost all of the produced NH_3_ species were used as the reactants for urea production. The FE and Y_urea_ of Co–NiO_x_@GDY remained almost unchanged before and after the stability test (Fig. 5g). Besides, detailed characterizations (e.g. SEM, TEM and EDX mapping measurements) on Co–NiO_x_@GDY obtained after long-time urea electrosynthesis showed no obvious changes in morphology and composition during the reaction, indicating the excellent stability of the catalyst (Supplementary Figs S28–S30). The isotopic labeling tests (Fig. 5h) using ^15^NO_2_^–^ as the N-source solidly demonstrated that the nitrogen in the synthesized urea originated from the nitrite in the electrolytes. The double-layer capacitance of Co–NiO_x_@GDY was 4.6 mF cm^–2^, which is larger than that of Co–NiO_x_ (2.5 mF cm^–2^) and the pure Ni foam (0.2 mF cm^–2^) (Fig. 5i)and Supplementary Figs S31–S33), revealing the largest electrochemically active surface area and further indicating the ideally engineered interface structure between Co–NiO_x_ and GDY with the best conductivity, the most facilitated charge-transfer kinetics, possessing essential advantages for efficient catalysis.

**Figure 6.**
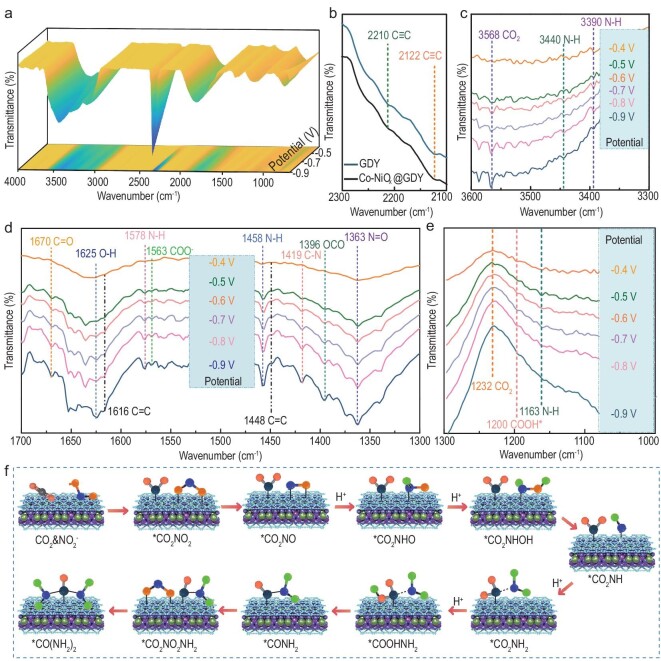
Operando SR-FTIR spectroscopy measurements under various potentials for Co–NiO_x_@GDY during electrocatalytic coupling of nitrate and carbon dioxide. (a) Three-dimensional FTIR spectra in the range of 4000–700 cm^−1^. (b) Experimental FTIR spectrum of GDY. (c) Infrared signals in the range of 3600–3300 cm^–1^. (d) Infrared signals in the range of 1700–1300 cm^–1^. (e) Infrared signals in the range of 1300–1000 cm^–1^. (f) Reaction mechanism studies for electrocatalytic urea synthesis on Co–NiO_x_@GDY.

In order to validate the C–N coupling mechanism of Co–NiO_x_@GDY, advanced operando SR-FTIR measurements were further carried out (Fig. 6a). Figure 6b)shows the typical FTIR spectrum of the GDY in which the peaks at 2122 and 2210 cm^–1^ originate from the typical C≡C stretching vibration. The same peaks of our Co–NiO_x_@GDY indicate the successful fabrication of the multi-heterointerface structure. The peak at 3568 cm^–1^ (Fig. 6c) indicates the CO_2_ adsorption on the catalysts and the peaks at 3440 and 3390 cm^–1^ indicate the formation of N–H during the reaction. The peak at 1670 cm^–1^ (Fig. 6d)and e) is assigned to the stretching of C=O and the peak at 1625 cm^–1^ is assigned to the O–H hydrogen bonding that seems to be due to the change in the adsorption configuration of water molecules after increasing the potential. Additionally, the peaks at 1578 and 1163 cm^–1^ belong to the bending mode and rocking mode of N–H, which indicates the formation of _*_NH_2_. And the peaks at 1419 and 1396 cm^–1^ reveal the presence of C–N and OCO, respectively [61–63]. An additional series of enhanced peaks at 1363 cm^–1^ represents the dissociated N=O obtained after adsorption on the Co–NiO_x_@GDY with increasing potential. The faint peak that appeared at 1200 cm^–1^ represents the adsorption of the intermediate _*_CO_2_NH_2_ with the hydroxyl. Compared with free urea, the shift in the stretching frequency for C–N implies that the produced urea interacted at the Co–NiO_x_@GDY surface via the O atoms in C=O. The overall urea electrosynthesis of the process involves four steps. First, the oxygen atoms in the nitrite electrolyte connect with the oxygen vacancy in Co–NiO_x_@GDY; second, the multi-step protons-couple occurs with the corresponding electron transfer to further form the important NH_2__*_ intermediate. Next, the CO_2_ molecules fill the vacancies in the Co–NiO_x_@GDY and are transferred to the COOH_*_ intermediate through a proton-coupled electron-transfer process; ultimately, the urea is formed by _*_CO_2_NH_2_ intermediates coupled from the NH_2__*_ and COOH_*_ (Fig. 6f) [64].

## CONCLUSIONS

Continuous synthesis of urea products under ambient conditions has not yet been realized by science and technology at present. Our study explored a sustainable urea production route using nitrite, carbon dioxide and water, and achieved high-performance synthesis under ambient conditions. Selective and active urea production with a record-high FE of 64.3%, N_urea_-selectivity of 86.0%, C_urea_-selectivity of ∼100%, urea yield rates of 913.2 μg h^–1^ mg_cat_^–1^ and excellent long-term stability on Co–NiO_x_@GDY was realized. Experimental results demonstrate that the *in situ* grown multi-heterojunction interfacial structure could lead to the formation of the strong incomplete charge-transfer phenomenon between a GDY and metal oxide interface and multiple intermolecular interactions. These effectively optimize the intermediate's adsorption/desorption abilities and promote direct C–N coupling by significantly suppressing by-product reactions toward the formation of H_2_, CO, N_2_ and NH_3_. Operando SR-FTIR results reveal the C–N coupling mechanism for urea synthesis. This work provides new insights into the design and synthesis of a multi-heterointerface catalyst for highly selective and efficient C–N coupling originating from NO_2_^−^ and CO_2_ under ambient conditions, which is a step forward towards the development of large-scale electrolysers.

## Supplementary Material

nwac209_Supplemental_File
