# Supplementary material for: Multi-heterointerfaces for selective and efficient urea production
Source: Natl Sci Rev. 2022 Oct 4;10(2):nwac209. doi: 10.1093/nsr/nwac209 (PMC9935990; doi:10.1093/nsr/nwac209)
Supplement: nwac209_Supplemental_File [file nwac209_supplemental_file.docx]

Supplementary Information for

**Multi-Heterointerfaces for Selective and Efficient Urea Production**

Danyan Zhang ^1,3^, Yurui Xue^1,2,^*, Xuchen Zheng^1,3^, Chao Zhang^1,3^, and Yuliang Li^1,2,^*

^1^Institute of Chemistry, Chinese Academy of Sciences, Beijing 100190, China;

^2^Science Center for Material Creation and Energy Conversion, Institute of Frontier and Interdisciplinary Science, School of Chemistry and Chemical Engineering, Shandong University, Jinan 250100, China. ^3^University of Chinese Academy of Sciences, Beijing 100049, China

***Corresponding authors.** E-mails: yrxue@sdu.edu.cn; ylli@iccas.ac.cn;

**Experimental Section**

**Materials.**

Co(NO_3_)_2_·6H_2_O and Ni(NO_3_)_2_·6H_2_O were brought from Energy. Tetrabutylammo-nium fluoride (TBAF) was purchased from Alfa Aesar. Hexabromobenzene was provided by J&K Scientific. All the other reagents were used as received. The water used in all experiments was purified with a Millipore system.

**Methods**

**Synthesis of Co-NiO_x_.**

The Co-NiO_x_H_y_ precursor was electrodeposited on a cleaned Ni Foam substrate by using an electrochemical workstation (Chenhua CHI760E, China). The washed Ni Foam was immersed in 50 mL of a solution containing 3 mmol Ni(NO_3_)_2_ ·6H_2_O and 6 mmol Co (NO_3_)_2_·6H_2_O as the working electrode. A saturated calomel electrode (SCE) was used as a reference electrode and a carbon rod as the counter electrode. The electrodeposition was performed under the potential of -1.1 V (vs. SCE) for 400 s. After electrodeposition, the sample was moved out and washed with distilled water and ethanol several times, then dried at 60 °C overnight. Next, the Ni Foam with the as-grown precursors was calcined at 300 °C for 3 h in the air to get the ultrathin porous Co-NiO_x_ nanosheet arrays.

**Synthesis of Co-NiO_x_@GDY.**

The GDY was prepared according to reported methods [32]. Five pieces of copper foil were added into the flask with 100 mL pyridine solution with Argon flow. The solutions of HEB (0.75 mg mL^−1^) were dripped into the flask. The cross-coupling reaction of HEB was carried out under the Argon atmosphere at 110 ^o^C in the dark. After 24 hours, the Co-NiO_x_ substrates with the in-situ growth of GDY nanosheets were treated with DMF, and acetone for several times. The Co-NiO_x_ @GDY positisample were obtained.

**Synthesis of NiO and CoO.**

The NiO, CoO were separately electrodeposited on a cleaned Ni Foam substrate immersed in 50 mL of a solution containing 9 mmol Ni(NO_3_)_2_ ·6H_2_O, 9 mmol Co(NO_3_)_2_ ·6H_2_O as the working electrode, respectively. The depositions were separately performed under the potential of -1.1 V (vs. SCE) for 400 s. After electrodeposition, the samples were separately moved out and washed with distilled water and ethanol several times, then dried at 60 °C overnight. Next, the as-grown precursors were separately calcined at 300 °C for 3 h in the air to get NiO, CoO nanosheet arrays.

**Synthesis of NiO@GDY and CoO@GDY.**

The GDY was prepared according to reported methods [32]. Five pieces of copper foil and as-prepared NiO, CoO nanosheet were separately added into the flask with 100 mL pyridine solution with Argon flow. The solutions of HEB (0.75 mg mL^−1^) were dripped into the flask containing NiO and CoO, respectively. The cross-coupling reaction of HEB was carried out under the Argon atmosphere at 110 ^o^C in the dark. After 24 hours, the NiO, CoO substrates with the in-situ growth of GDY nanosheets were treated with DMF, and acetone for several times. The NiO@GDY and CoO@GDY sample were obtained.

**Determination of urea.**

The produced urea has been spectrophotometrically detected by the diacetylmonoxime method. 1 ml of the solution was firstly removed from the H-type cell. Secondly, 2 ml acid-ferric solution which contains 100 ml concentrated phosphoric acid, 300 ml concentrated sulfuric acid, 600 ml deionized water and 100 mg ferric chloride and 1 ml diacetylmonoxime (DAMO)-thiosemicarbazide (TSC) solution were added. (The diacetylmonoxime (DAMO)-thiosemicarbazide (TSC) solution : 5 g DAMO and 100 mg TSC were dissolved in 1000 ml deionized water.) The urea-containing solution was heated to 110 °C and kept for 15 min. After cooling down, the UV-vis absorbance were measured at 525 nm using a UV–Vis spectrophotometer. The concentrations–absorbance curves were calibrated using standard urea solutions (Supplementary Fig. S34, available as Supplementary Data at NSR online).

**Determination of ammonia.**

The produced ammonia was spectrophotometrically determined by the indophenol blue method. 2 ml of the solution was removed from the H-type cell firstly. Secondly, 2 ml of a 1 M NaOH solution which chontains 5 wt% salicylic acid and 5 wt% sodium citrate was added. Next,1 ml of 0.05 M NaClO and 0.2 ml of an aqueous solution of 1wt% C_5_FeN_6_Na_2_O (sodium nitroferricyanide) were added. After 2 h in the dark, the absorption spectra was measured using a UV– Vis spectrophotometer. The concentration of indophenol blue was determined using UV-vis absorbance at a wavelength of 655 nm. The concentrations–absorbance curves were calibrated using standard ammonia sulfate solutions (Supplementary Fig. S35, available as Supplementary Data at NSR online).

**Determination of nitrite ions (NO_2_**^–^**).**

The amounts of possible NO_2_^–^ were quantified by the N-(-1-naphthyl)-ethylenediamine dihydrochloride method. Firstly, 0.5 g of sulfanilic acid was dissolved in 90 mL H_2_O and 5 mL acetic acid. Secondly, 5 mg of n-(1-naphthyl)-ethylenediamine dihydrochloride was added and then the solution was filled to 100 mL. Next, 1 ml of the treated electrolyte mixing with 4 mL of chromogenic agent, which was subsequently kept in dark for 15 min. The UV-Vis absorption spectrum was then acquired at 540 nm. The concentration–absorbance curves were calibrated using standard ammonia sulfate solutions (Supplementary Fig. S36, available as Supplementary Data at NSR online).

**Determination of carbon monoxide (CO) and hydrogen (H_2_).**

H_2_ and CO were quantified by GC (Agilent 7890A) equipped with a thermal conductive detector (TCD) using Ar as the carrier gas for H_2_ quantification and He as the carrier gas for CO detection.

**Calculations of average urea yield rate and FE.**

The average yield rate of urea was calculated by the formula:

Y_urea_ = (c_urea_ × V) / (t × m)

c_urea_ is the measured urea concentration (μg ml^–1^), V is the total volume of electrolyte (ml), t is time (h) for electrocatalysis and m is the catalyst loadings (mg). The FE for urea electrosynthesis can be calculated by the formula:

FE = (e × F × c_urea_ × V) / (60.06 × Q)

e is the valence change for reactant, F is the Faraday constant (96,485.3 C mol^–1^) and *Q* is the total charge passed through the working electrode (C).

**Calculations of average ammonia yield rate and FE.**

The average yield rate of ammonia was calculated by the formula:

Y_NH3_ = (c_NH3_ × V)/ (t × m)

c_NH3_ is the measured NH_3_ concentration (μg ml^–1^), V is the total volume of electrolyte (ml), t is time (h) and *m* is the catalyst loadings (mg).

The FE for NO_2_^–^RR can be calculated by the formula:

FE = (e × F × c_NH3_ × V) / (17 × Q)

e is the valence change for reactant, F is the Faraday constant (96,485.3 C mol^–1^) and Q is the total charge passed through the working electrode (C).

**Calculations of N_urea_-selectivity**

The N_urea_-selectivity was calculated by the formula:

N_urea_ - selectivity = n_urea_ (N) /n_total_ (N)

the n_urea_ (N) represents the moles of nitrogen in urea and total (N) represents the moles of nitrogen in total products from nitrite.

**Characterizations**

Scanning electron microscopy (SEM) images were collected using an S-4800 field emission scanning electron microscope. Transmission electron microscopy (TEM) and high-resolution TEM (HRTEM) images were recorded on a JEM-2100F electron microscope operating at 200 kV. X-ray diffraction (XRD) was conducted on a Japan Rigaku D/max-2500 rotation anode X-ray diffractometer using Cu Kα radiation (λ = 1.54178 Å). Raman spectra were obtained by a Renishaw-2000 Raman spectrometer exploiting a 514.5 nm excitation laser source. The X-ray photoelectron spectroscopy (XPS) data were obtained by a Thermo Scientific ESCALab 250Xi instrument with monochromatic Al Kα X-ray radiation.

**Electrochemical urea synthesis.**

The Nafion 117 membrane was firstly pretreated by heating it in H_2_O_2_ (5%) aqueous solution at 80 °C for 1 h and DI-water at 80 °C for 1 h, respectively, followed by treated in 0.05 M H_2_SO_4_ for 1 h and DI-water for another 3 h. All electrochemical experiments in this study were tested using a typical three-electrode system by an electrochemical workstation (CHI. 660D, Shanghai CH. Instruments, China). The catalysts were employed as a working electrode; the graphite rod and saturated calomel electrode (SCE) were used as the counter electrode and the reference electrode, respectively. We used 0.01 M NaNO_2_ (saturated with CO_2_) as the electrolyte to reduce the impact of CO_2_ solubility. All potentials were converted to RHE. For electrochemical urea synthesis, potentiostatic tests were carried out in CO_2_-saturated 0.01 M NaNO_2_, which was bubbled with CO_2_ for 20 min before the measurement. Then the purified CO_2_ was continuously fed into the cathodic compartment with a constant flow rate of 20 ml min^-1^ during the experiments.


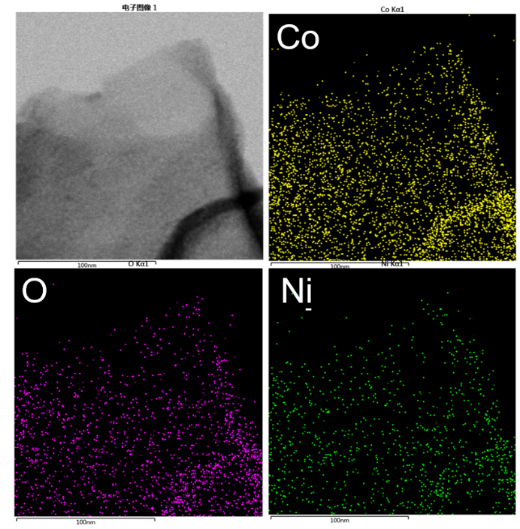


**Supplementary Figure S1.** EDX mapping of Co-NiO_x_H_y_.


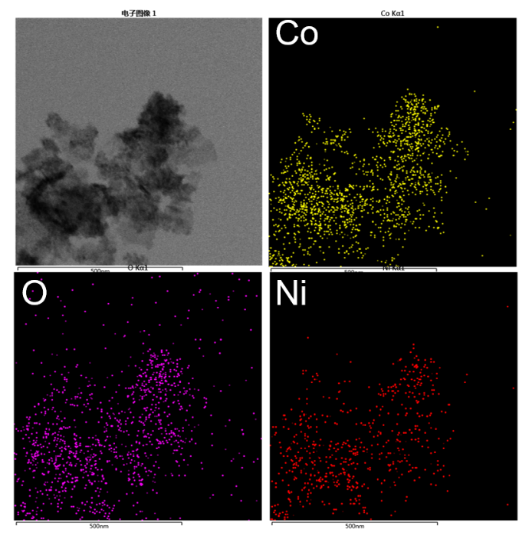


**Supplementary Figure S2**. TEM images of Co-NiO_x._


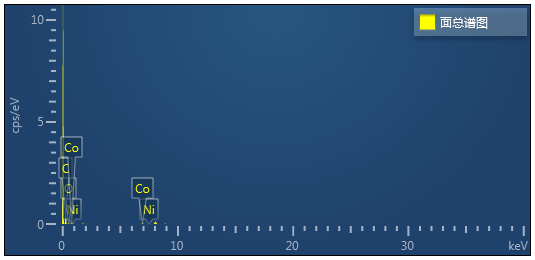


**Supplementary Figure S3**. The eds spectra of the elemental distribution surface on Co-NiO_x_@GDY.


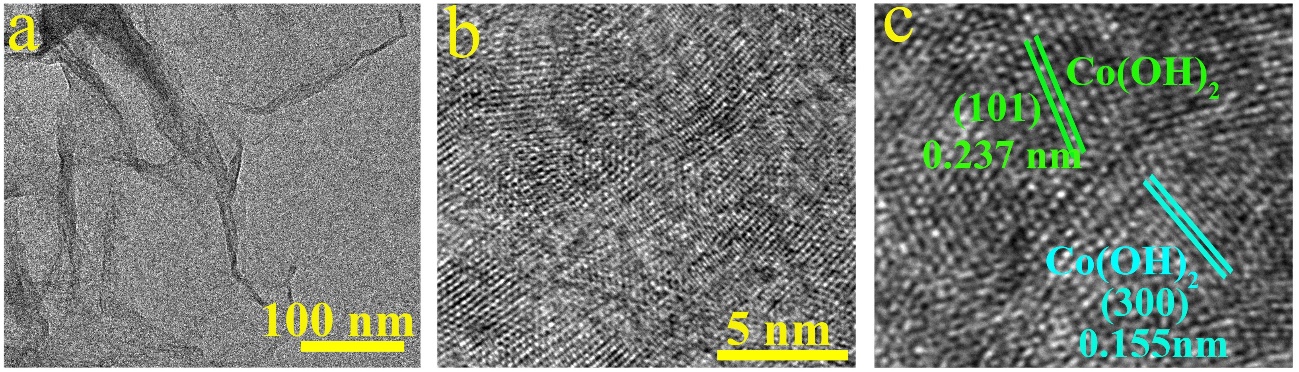


**Supplementary Figure S4.** TEM images of Co-NiOxHy_._

_
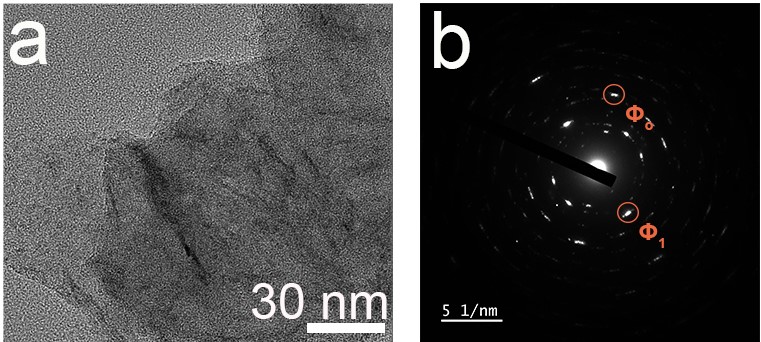
_

**Supplementary Figure S5.** (a) The bright field TEM image and (b) the diffraction pattern of Co-NiO_x_H_y_.

_
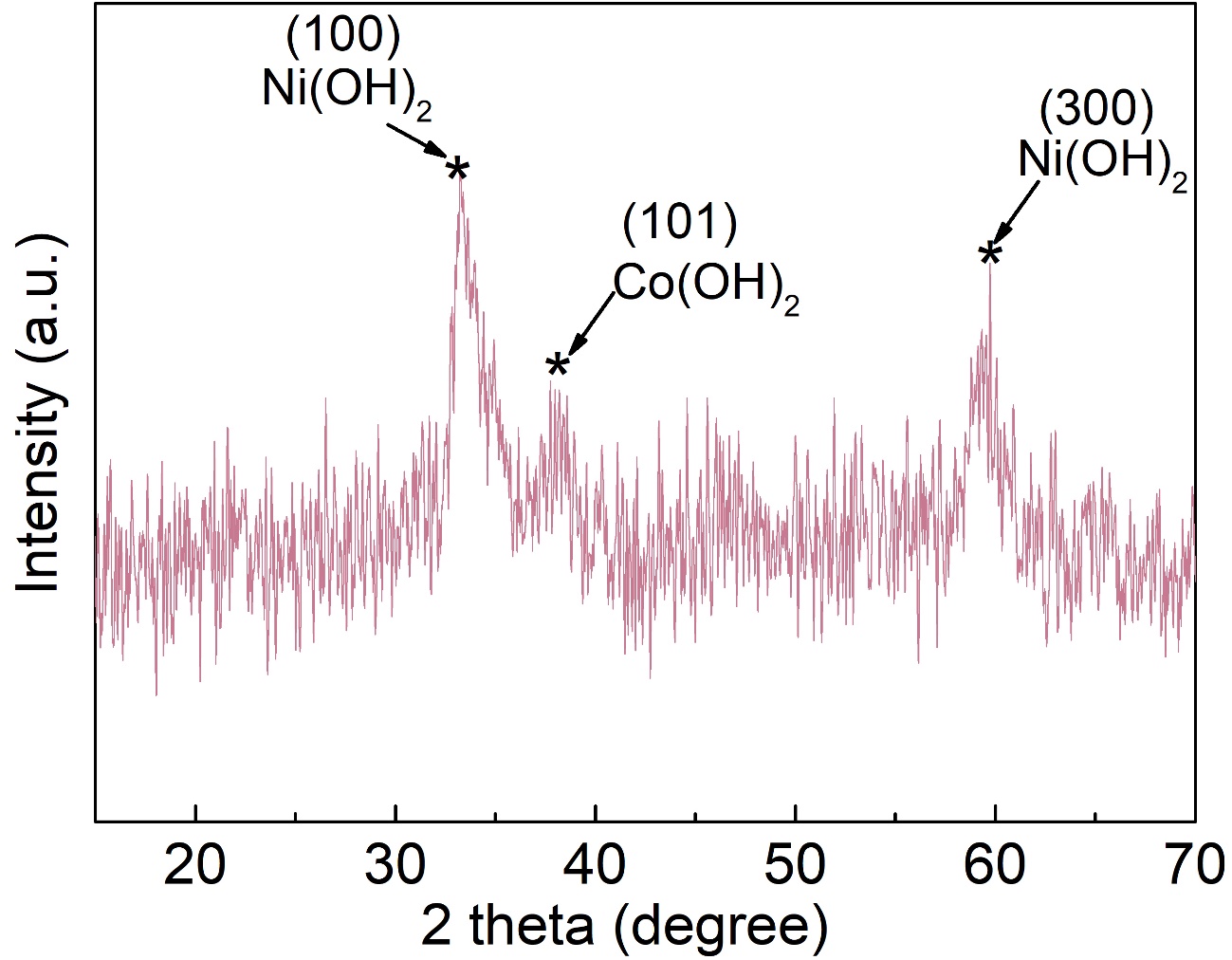
_

**Supplementary Figure S6.** The powder XRD patterns of Co-NiO_x_H_y_.


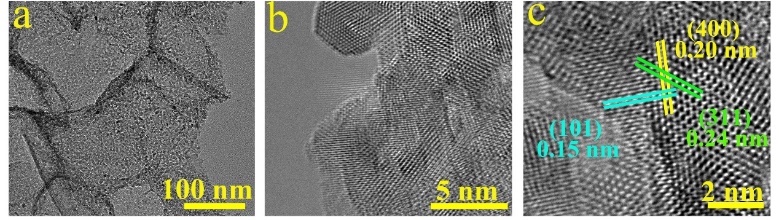


**Supplementary Figure S7.** TEM images of Co-NiO_x._

**
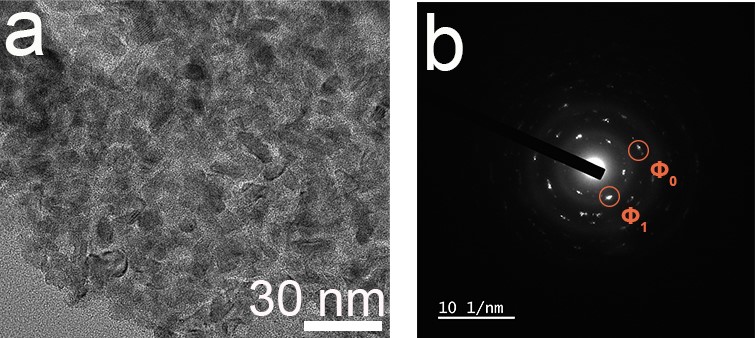
**

**Supplementary Figure S8.** (a) The bright field TEM image and (b) the diffraction pattern of Co-NiO_x_.

**
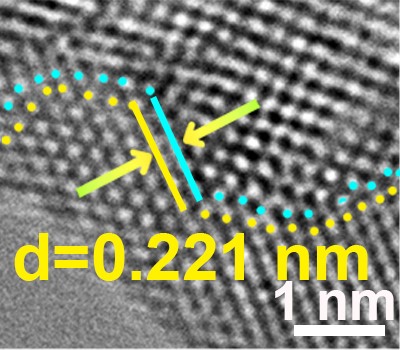
**

**Supplementary Figure S9.** The distortion of the atomic layers at the interface of Co-NiO_x_.


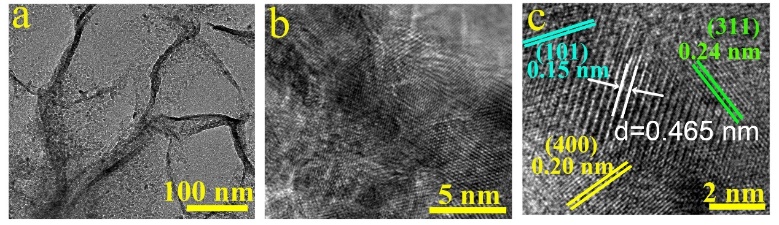


**Supplementary Figure S10.** TEM images of Co-NiO_x_ @GDY_._

_
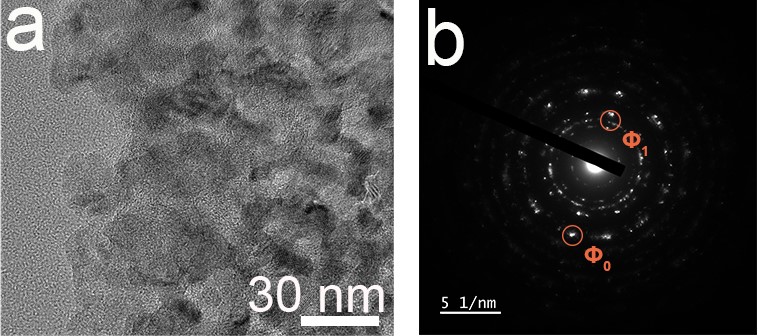
_

**Supplementary Figure S11.** (a) The bright field TEM image and (b) the diffraction pattern of Co-NiO_x_@GDY.


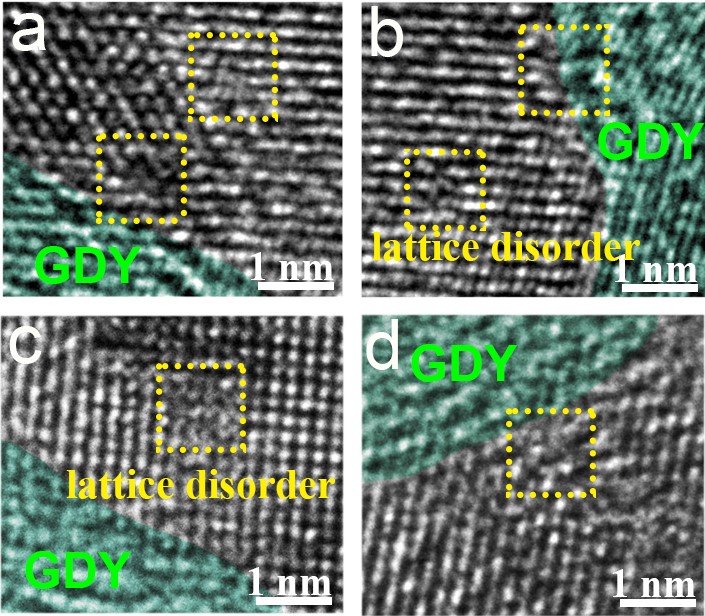


**Supplementary Figure S12.** Lattice disorder in Co-NiO_x_ @GDY.


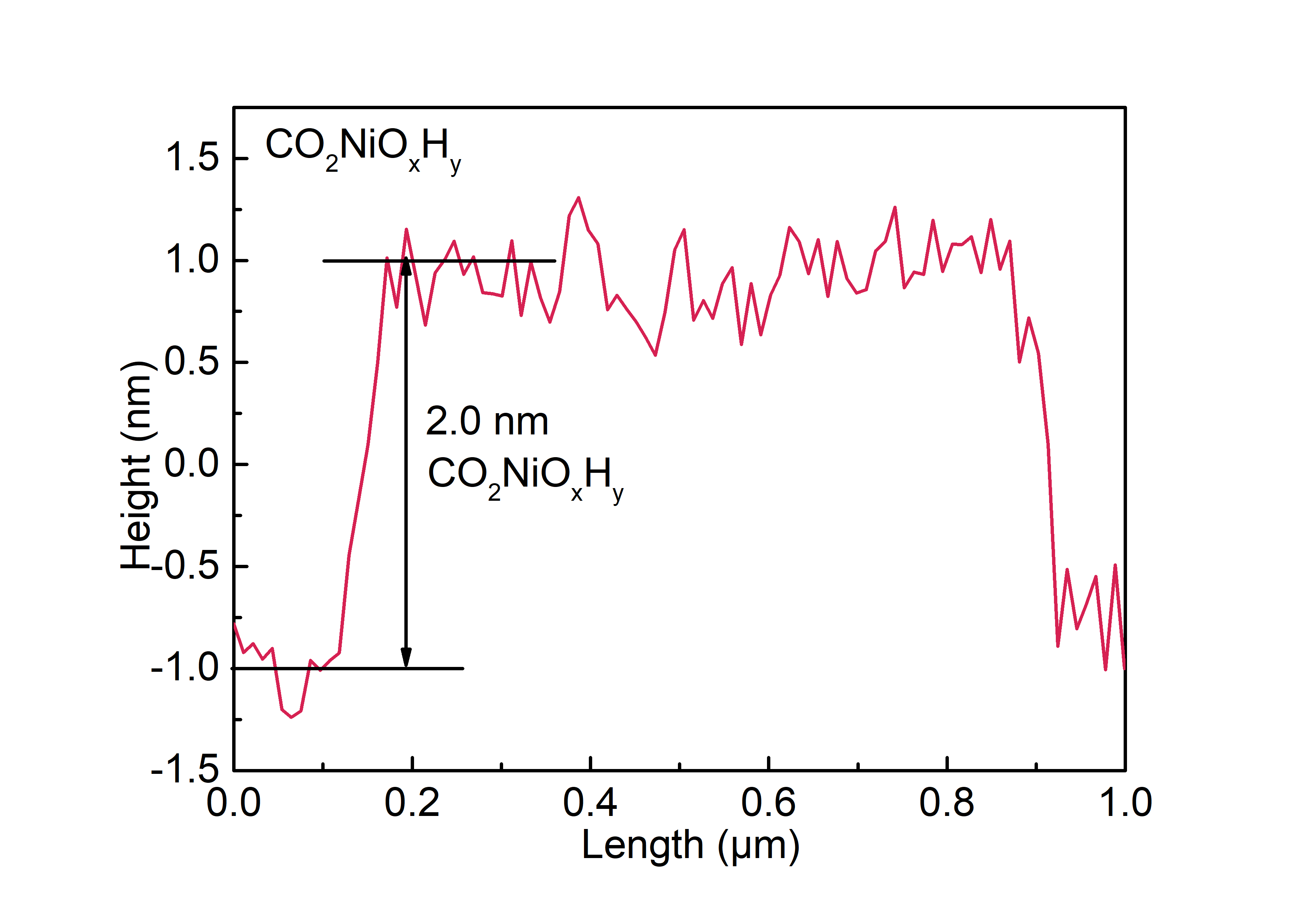


**Supplementary Figure S13.** Thickness measurement of Co-NiO_x_H_y_ nanosheets.


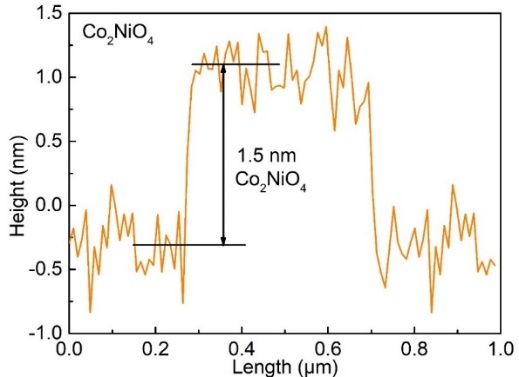


**Supplementary Figure S14.** Thickness measurement of Co-NiO_x_ nanosheets.


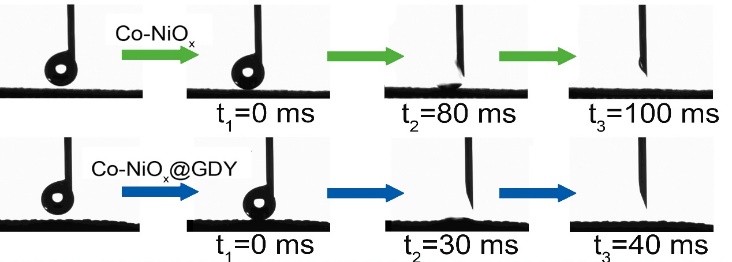


**Supplementary Figure S15.** Contact angle measurements of Co-NiO_x_ and Co-NiO_x_@GDY, respectively.


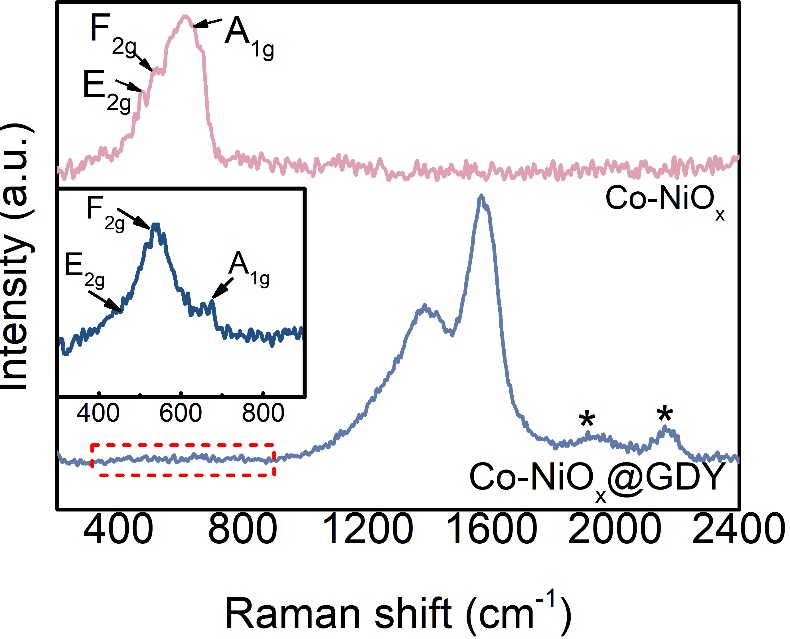


**Supplementary Figure S16.** The magnified Raman spectra of Co-NiO_x_@GDY.

**
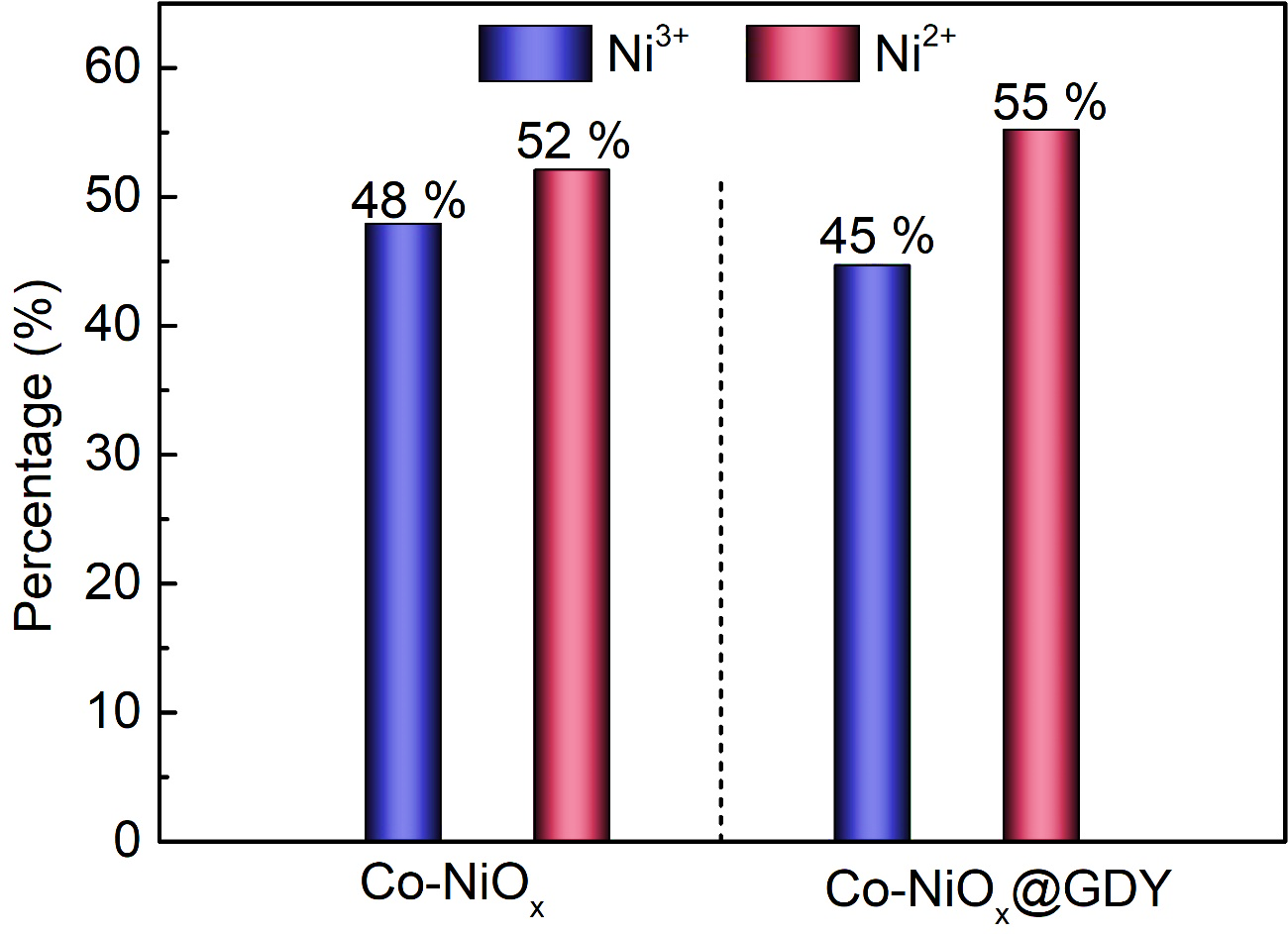
**

**Supplementary Figure S17.** The percentage of Ni^2+^ and Ni^3+^ species in Co-NiO_x_ and Co-NiO_x_@GDY.


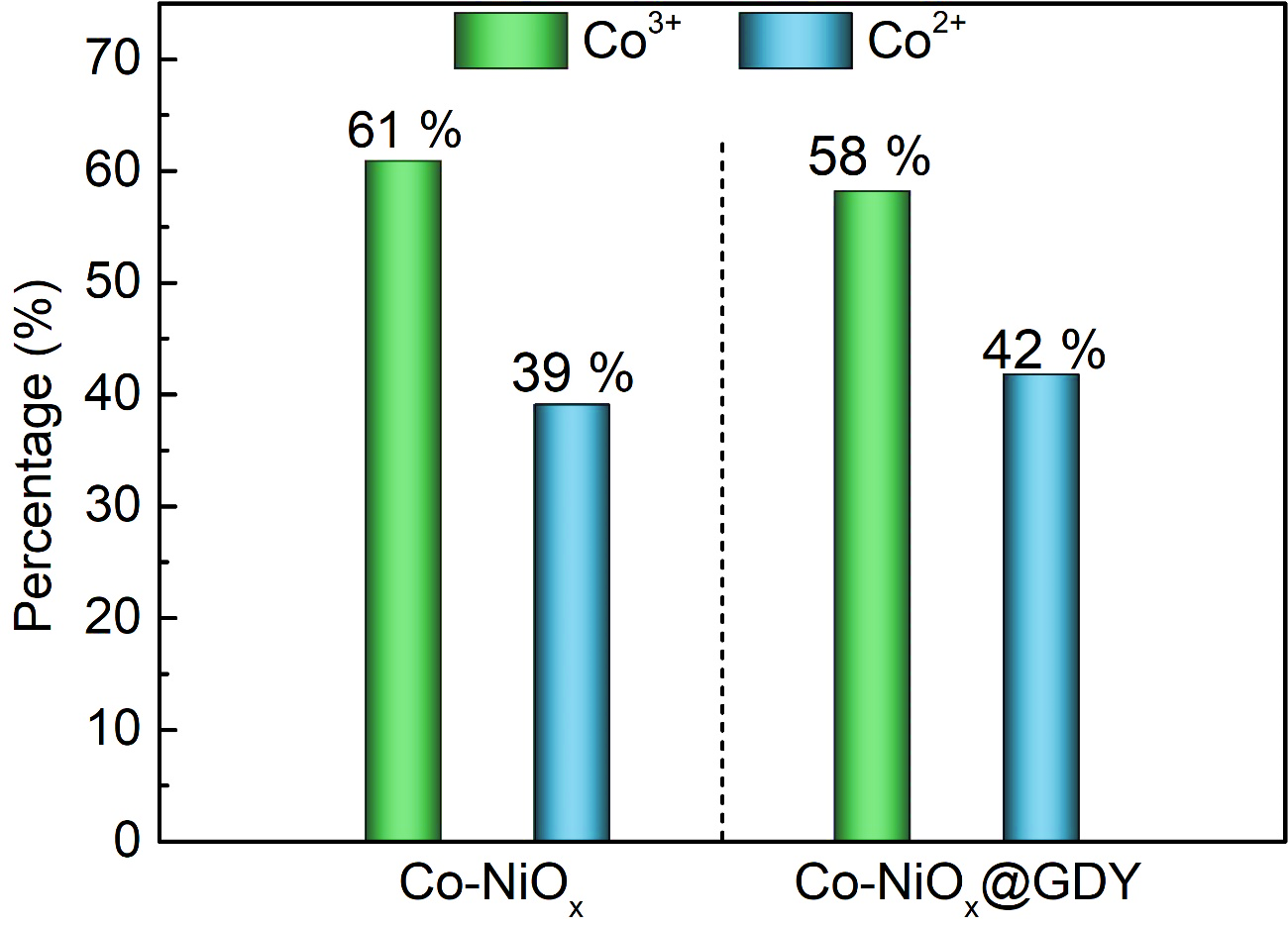


**Supplementary Figure S18.** The percentage of Co^2+^ and Co^3+^ species in Co-NiO_x_ and Co-NiO_x_@GDY.


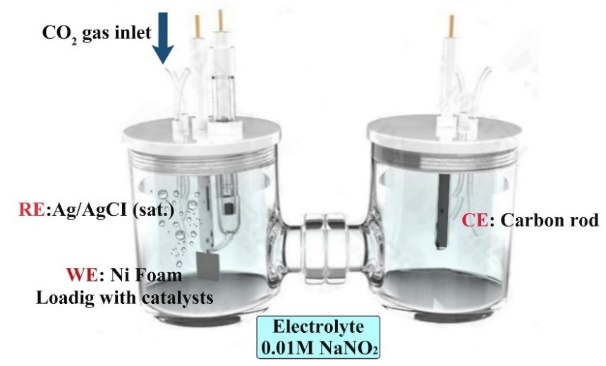


**Supplementary Figure S19.** Schematic diagram of the H-type cell (H-cell) for urea synthesis.


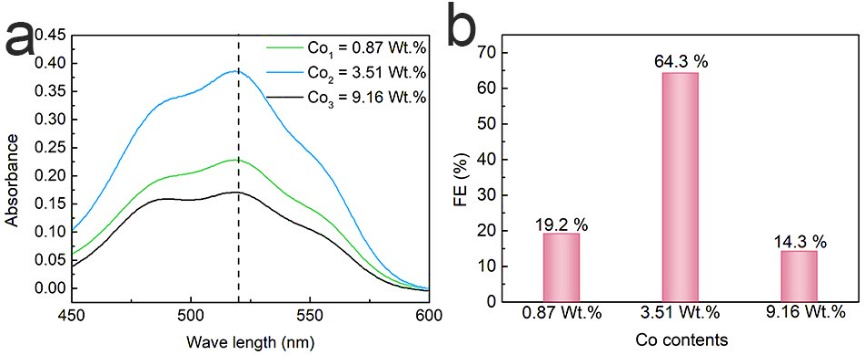


**Supplementary Figure S20.** (a) UV curves and (b) FE with different Co contents for urea synthesis.


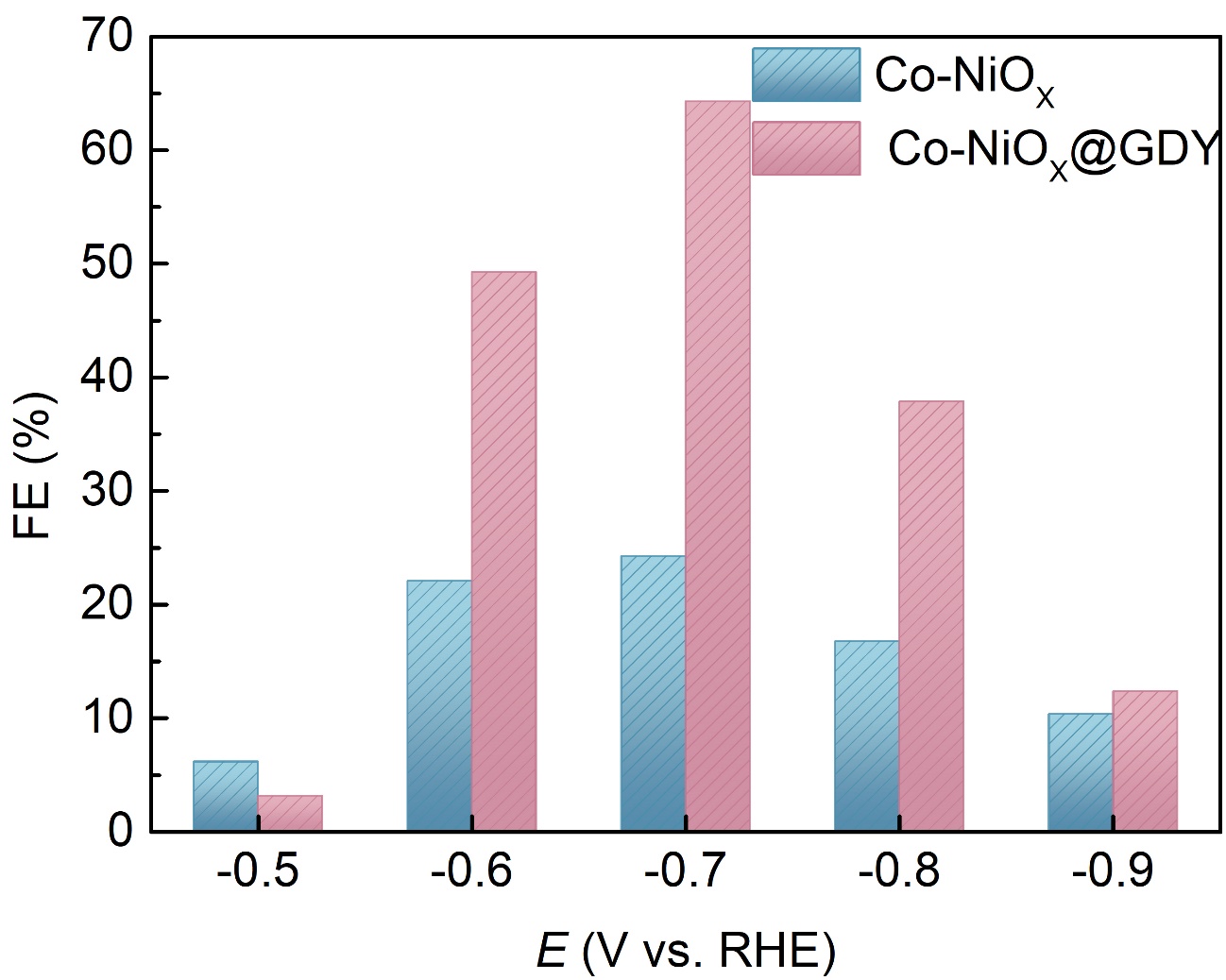


**Supplementary Figure S21.** Urea FE comparison of Co-NiO_x_ and Co-NiO_x_ @GDY.

**
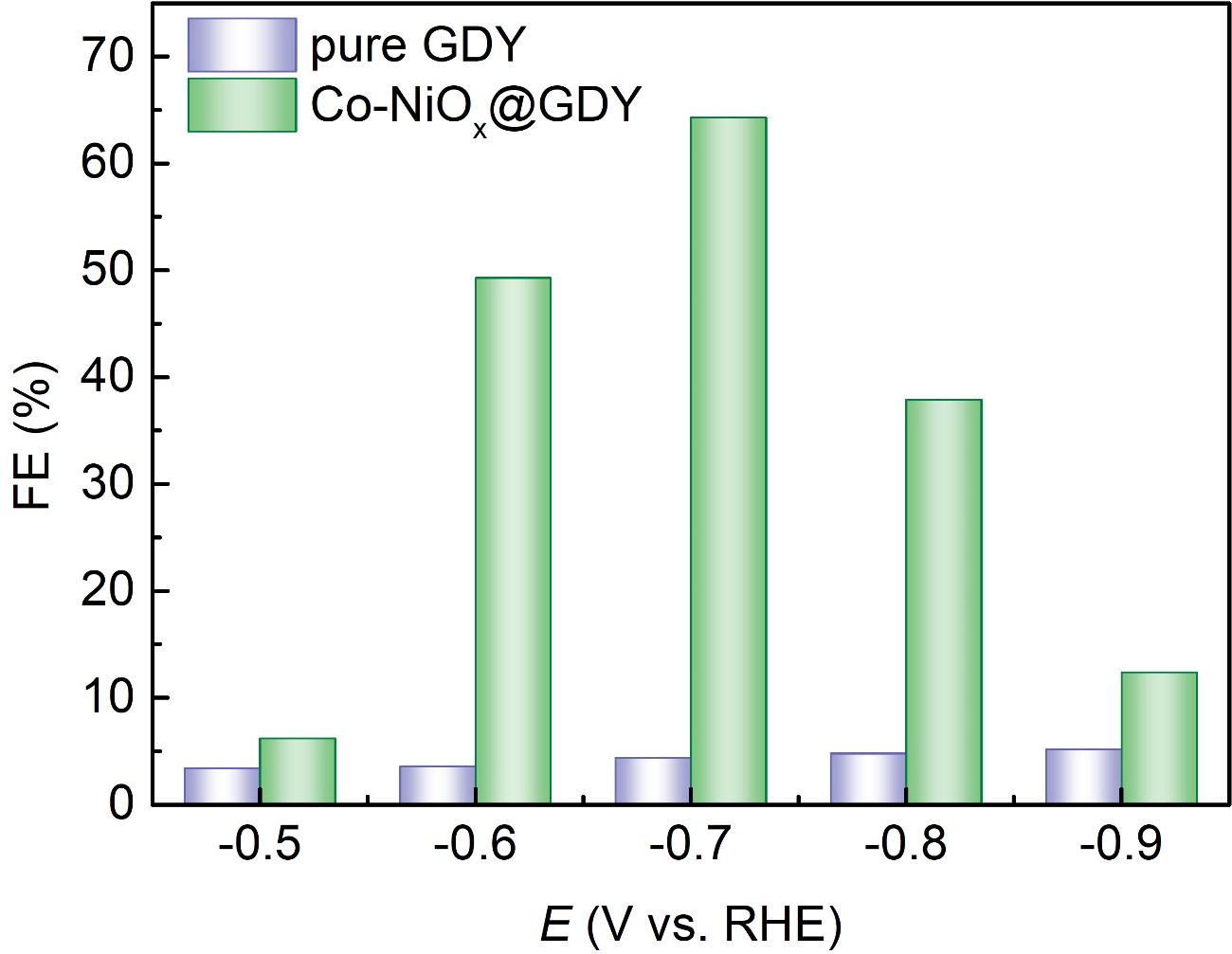
**

**Supplementary Figure S22.** FE comparison on urea synthesis of pure GDY and Co-NiO_x_@GDY.


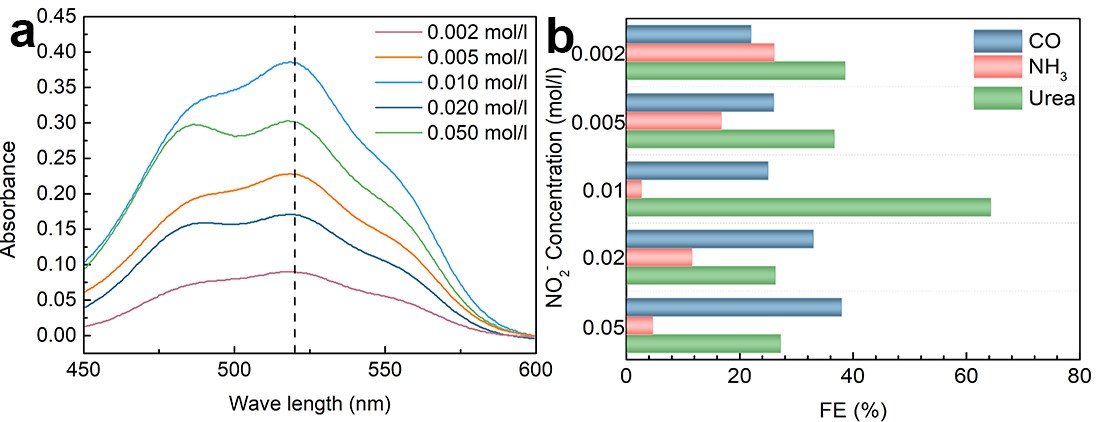


**Supplementary Figure S23.** (a) UV curves of Co-NiO_x_@GDY for urea synthesis under different concentrations of NO_2_^-^. (b) FE of the urea, CO, and NH_3_ at different concentrations of NO_2_^-^ for urea synthesis.

**
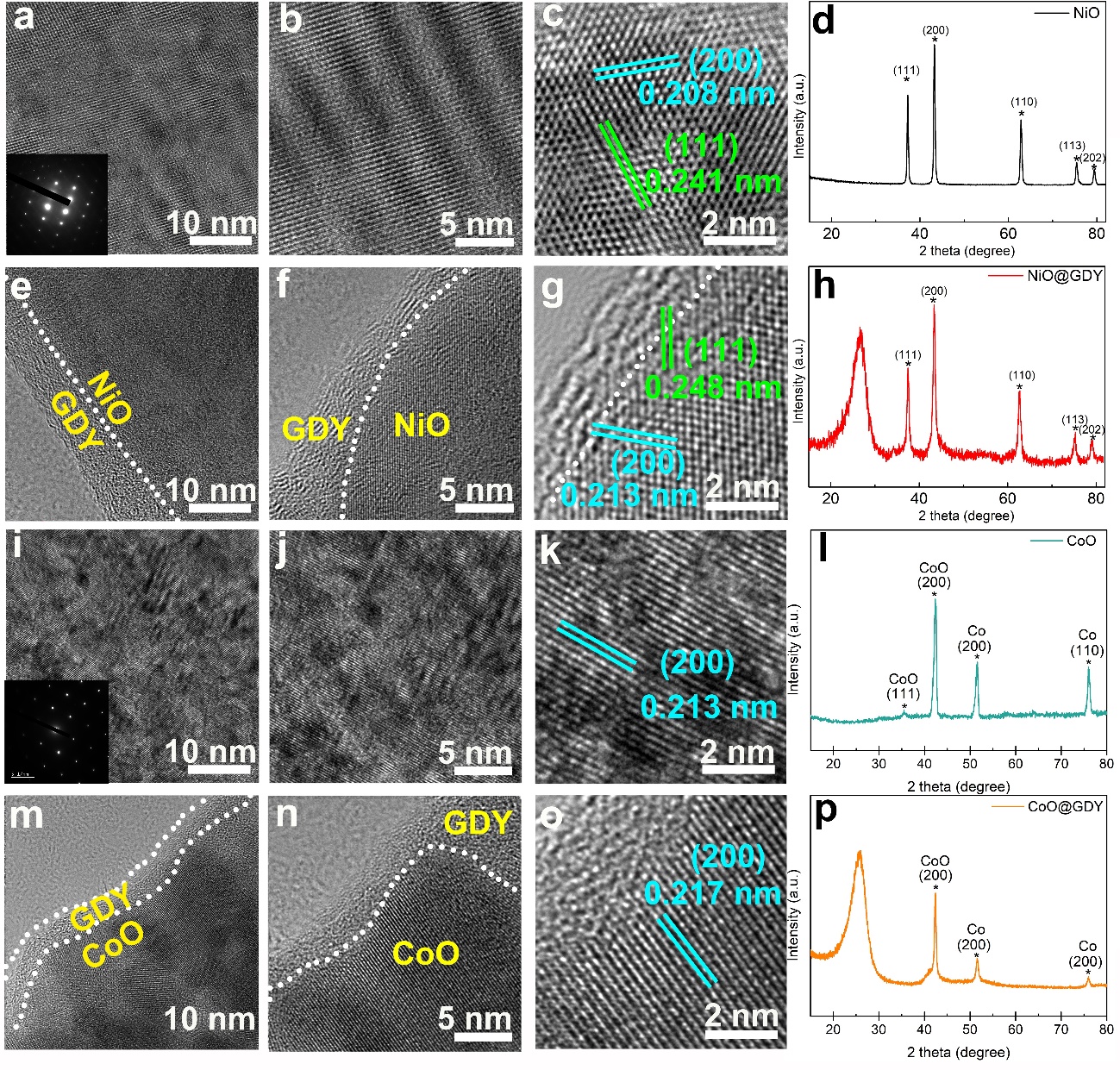
**

**Supplementary Figure S24.** (a-b) The TEM images of NiO. (c) The HRTEM image of NiO. (d) The powder XRD of NiO. (e-f) The TEM images of NiO@GDY. (g) The HRTEM image of NiO@GDY. (h) The powder XRD of NiO@GDY. (i-j) The TEM images of CoO. (k) The HRTEM image of CoO. (l) The powder XRD of CoO. (m-n) The TEM images of CoO@GDY. (o) The HRTEM image of CoO@GDY. (p) The powder XRD of CoO @GDY.


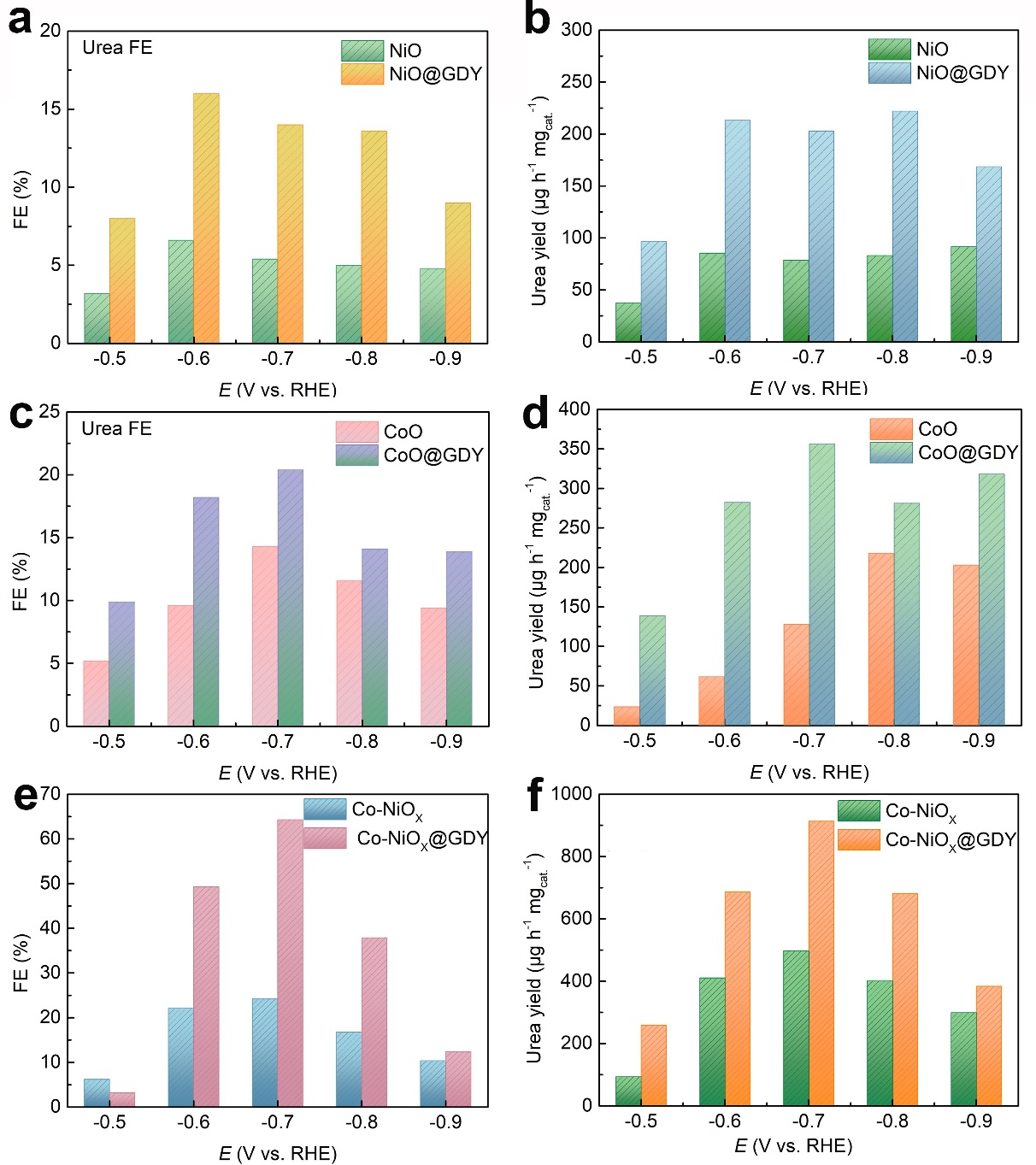


**Supplementary Figure S25.** (a) FE and (b) Urea yield of NiO and NiO@GDY obtained at different potentials. (c) FE and (d) Urea yield of CoO and CoO@GDY obtained at different potentials. (e) FE and (f) Urea yield of Co-NiO_X_ and Co-NiO_X_ @GDY obtained at different potentials.


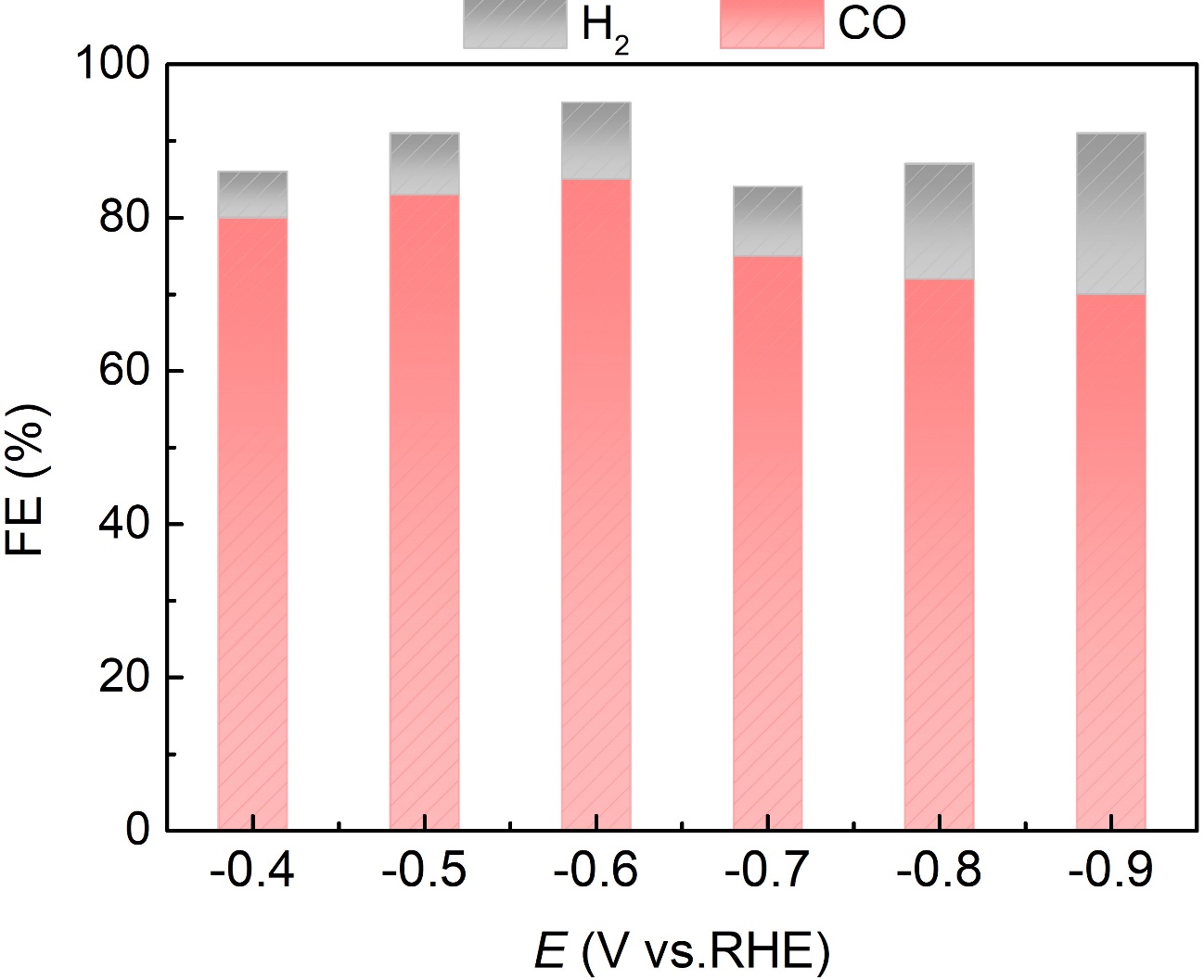


**Supplementary Figure S26.** CO and H_2_ FE on Co-NiO_x_ @GDY in CO_2_-saturated 0.01 M NaHCO_3_.


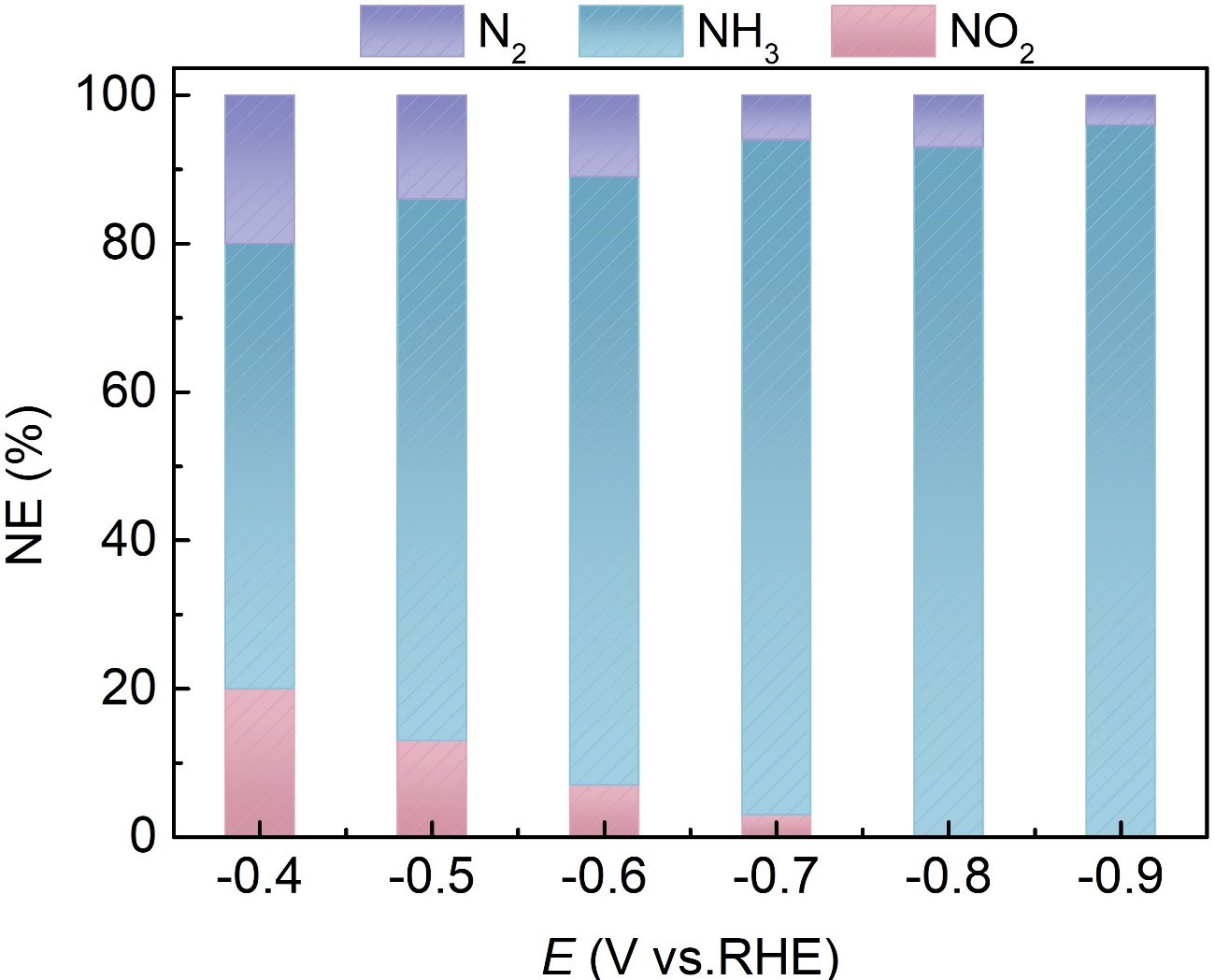


**Supplementary Figure S27.** NO_2_^-^, N_2_, and NH_3_ NE on NE on Co-NiO_x_ @GDY.


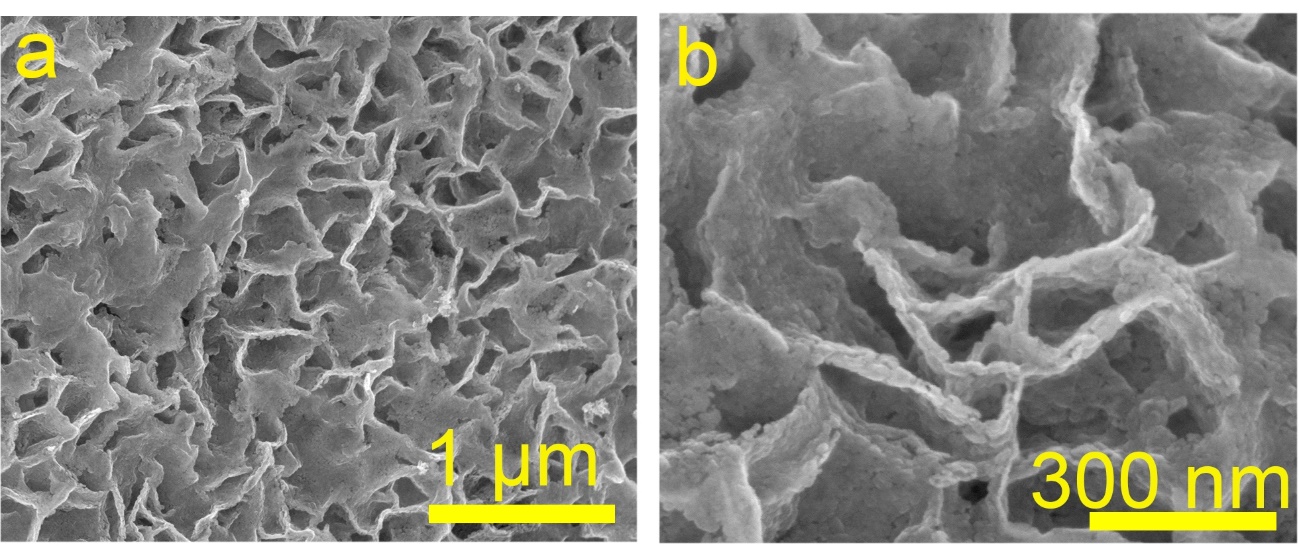


**Supplementary Figure S28.** SEM images of Co-NiO_x_ @GDY after the electrochemical test.


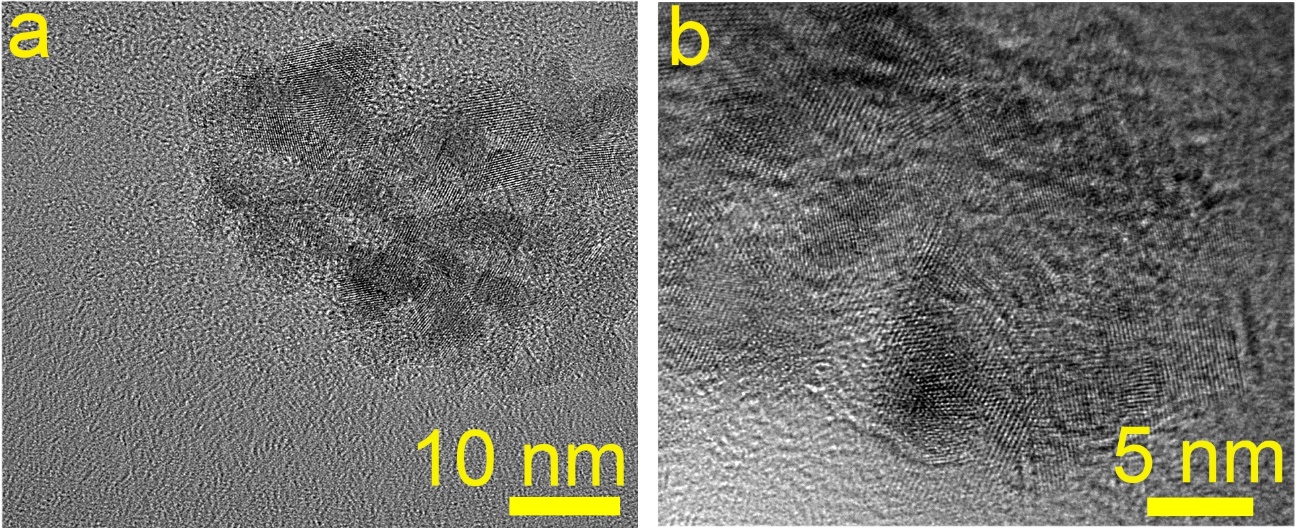


**Supplementary Figure S29.** TEM images of Co-NiO_x_ @GDY after the electrochemical test.


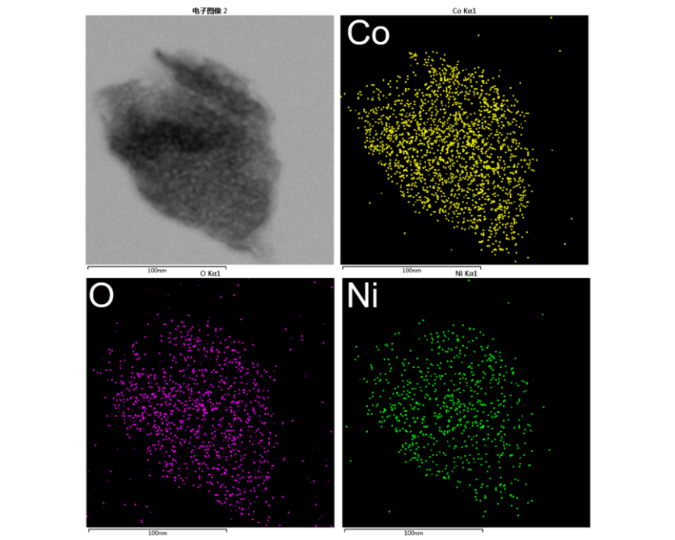


**Supplementary Figure S30.** EDS mapping of Co-NiO_x_ @GDY after the electrochemical test.


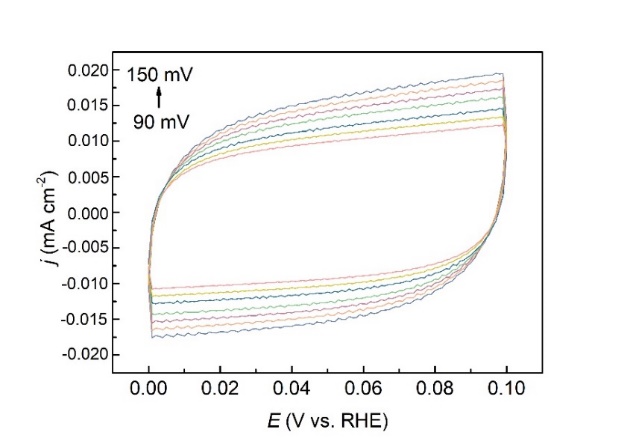


**Supplementary Figure S31.** The C_dl_ curves of NF.


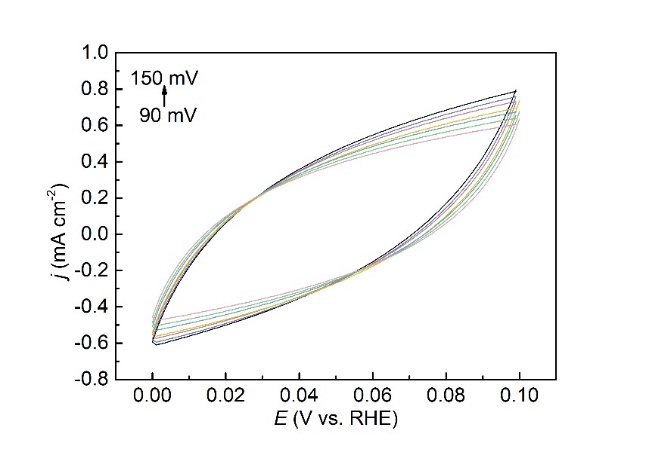


**Supplementary Figure S32.** The C_dl_ curves of Co-NiO_x_.


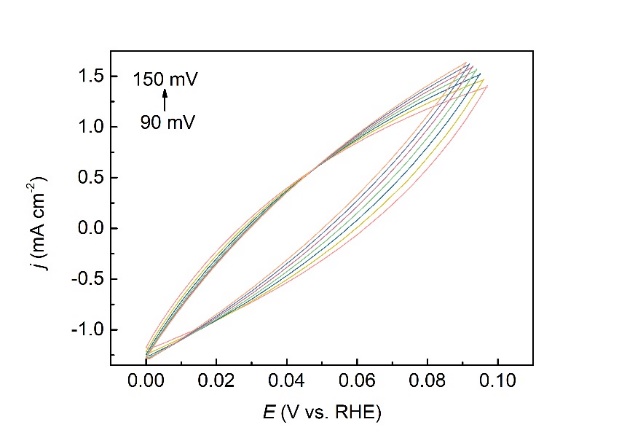


**Supplementary Figure S33.** The C_dl_ curves of Co-NiO_x_ @GDY.


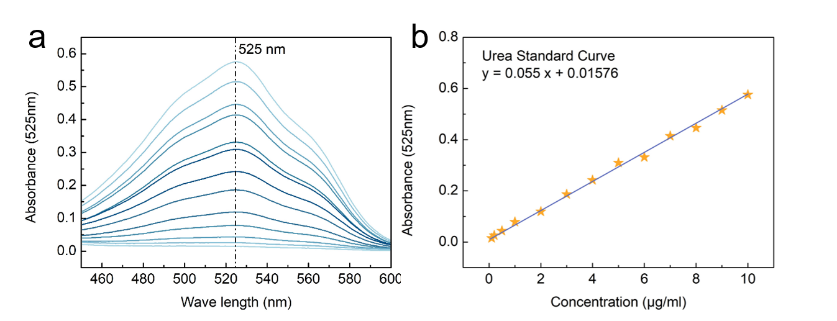


**Supplementary Figure S34.** The quantification of urea concentration.


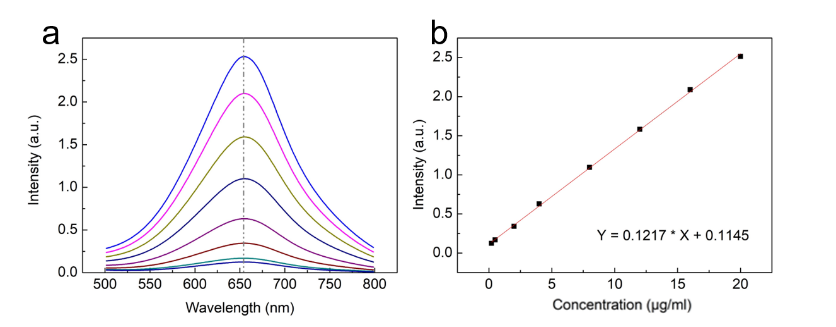


**Supplementary Figure S35.** The quantification of NH_3_ concentration.


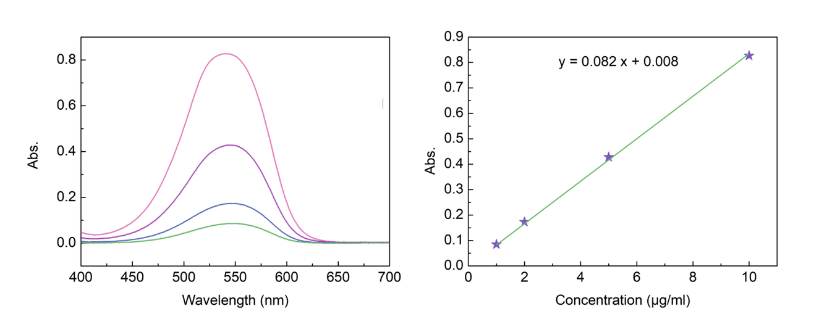


**Supplementary Figure S36.**The quantification of NO_2_^-^ concentration.
